# Meniscus-assisted solution printing of large-grained perovskite films for high-efficiency solar cells

**DOI:** 10.1038/ncomms16045

**Published:** 2017-07-07

**Authors:** Ming He, Bo Li, Xun Cui, Beibei Jiang, Yanjie He, Yihuang Chen, Daniel O’Neil, Paul Szymanski, Mostafa A. EI-Sayed, Jinsong Huang, Zhiqun Lin

**Affiliations:** 1 School of Materials Science and Engineering, Georgia Institute of Technology, Atlanta, Georgia 30332, USA; 2 Laser Dynamics Laboratory, School of Chemistry and Biochemistry, Georgia Institute of Technology, Atlanta, Georgia 30332, USA; 3 Department of Mechanical and Materials Engineering, University of Nebraska–Lincoln, Lincoln, Nebraska 68588, USA

## Abstract

Control over morphology and crystallinity of metal halide perovskite films is of key importance to enable high-performance optoelectronics. However, this remains particularly challenging for solution-printed devices due to the complex crystallization kinetics of semiconductor materials within dynamic flow of inks. Here we report a simple yet effective meniscus-assisted solution printing (MASP) strategy to yield large-grained dense perovskite film with good crystallization and preferred orientation. Intriguingly, the outward convective flow triggered by fast solvent evaporation at the edge of the meniscus ink imparts the transport of perovskite solutes, thus facilitating the growth of micrometre-scale perovskite grains. The growth kinetics of perovskite crystals is scrutinized by *in situ* optical microscopy tracking to understand the crystallization mechanism. The perovskite films produced by MASP exhibit excellent optoelectronic properties with efficiencies approaching 20% in planar perovskite solar cells. This robust MASP strategy may in principle be easily extended to craft other solution-printed perovskite-based optoelectronics.

## Abstract (web summary)

The morphology control of metal halide perovskite crystalline films is of importance to enable high-performance solar cells. Here, He *et al*. use a meniscus-assisted solution-based method to print microsized perovskite grains at 60 °C, which results in high optoelectronic device efficiency of 20%.

Solution printing is widely recognized as an attractive route to cost-effective, high-throughput and large-area thin film optoelectronics^1^,^2^,^3^. In this technique, the ink solution containing semiconductor precursors or nanoparticles can be readily deposited on a variety of desirable substrates, offering precise control over stoichiometry and rendering ubiquitous adaptability and compatibility with patterned and flexible substrates^4^. As state-of-the-art direct bandgap semiconductors, metal halide perovskites capitalize on superior optoelectronic characteristics such as intense broadband absorption, large ambipolar mobility, long charge carrier lifetime, and low processing cost compared with inorganic counterparts (e.g., silicon, CIGS and CdTe)^5^,^6^. The power conversion efficiency (PCE) of solution-processed metal halide perovskite solar cells has rapidly leapfrogged from 3.8% to 22.1% over the past several years^7^,^8^. Notably, top-performing perovskite solar cells have recently been advanced largely by optimizing the crystal morphology of methylammonium lead halides (MAPbX_3_, X=I, Br and Cl), formamidinium lead halides (FAPbX_3_), and their combinations^9^,^10^,^11^. However, the requirement of morphology control has posed a great challenge for solution-printed perovskite films due to the limited understanding of crystallization in dynamic flow of perovskite inks.

It is important to note that photoluminescence spectroscopy has revealed that the trap-assisted non-radiative charge recombination dominates the efficiency loss in perovskite solar cells^12^,^13^. The existence of charge traps is strongly associated with the disorder of crystals caused by ionized impurities, lattice vacancies and interstices and grain-boundary defects within perovskite film^14^. Through improving the crystallization, charge traps inside high-crystallinity perovskites exist at shallow states, yet deep traps are still massively present near the grain boundary region^15^. Recently, several solution-processed routes to micrometre-sized perovskite grains by crafting films at high temperatures (for example, spin coating at 180 °C for MAPbI_3(1−*x*)_Cl_3*x*_ and doctor-blading at 145 °C for FA_*x*_MA_1−*x*_PbI_3_, 0≤*x*≤1) have been successfully implemented to reduce the areas of grain boundary and minimize the trap-induced energy loss^16^,^17^. One notion behind these effective approaches is that the grain-boundary motion depends strongly on the temperature, and a high temperature can stimulate solute atoms more easily to overcome the diffusion energy barrier, thus resulting in fast grain growth rate^18^,^19^. Nevertheless, solution printing may not be amenable to high-temperature preparative conditions that are close to the boiling point, *T*_bp_, of typical solvents used for dissolving perovskites (for example, dimethylformamide (DMF), *T*_bp, DMF_=∼152 °C; dimethyl sulfoxide (DMSO), *T*_bp, DMSO_=∼189 °C), as the ink solution is too volatile to render the control over the crystal morphology. In addition to increased energy consumption, high-temperature treatment is also likely to cause thermal degradation and thermo-mechanical fatigue of perovskite and electrode materials, especially for flexible electrodes such as polyethylene terephthalate (PET) and polyethylene naphthalate (PEN)^20^. Clearly, a low-temperature strategy for morphological control of solution-printed perovskite films is highly desirable, which has yet to be explored.

Here we report on a versatile meniscus-assisted solution printing (MASP) strategy to create highly crystallized perovskite films with micrometre-scale grains and preferred crystal orientations at low temperatures for high-efficiency perovskite solar cells. Specifically, a meniscus is formed due to capillary action by constraining the perovskite ink between a lower substrate and an upper plate. The fast solvent evaporation at the edge (that is, the air/perovskite ink/lower substrate three-phase contact line) of the meniscus triggers an outward convective flow to replenish the evaporative loss of solvent and meanwhile transports the perovskite solutes toward the contact line due to the coffee ring effect^21^,^22^,^23^. This yields a perovskite supersaturated phase containing continually aggregated solutes, promoting the nucleation and growth of perovskite crystals. Through placing the lower substrate on a programmed translational stage, the receding meniscus can be slowly and progressively swept across the entire lower substrate, thereby printing a continuous perovskite film on the substrate. We note that the MASP deposition is inspired by solution-coating techniques in the field of organic semiconductor processing^24^,^25^,^26^,^27^. For solution-based coating of metal halide perovskite materials, doctor-blade coating has been widely employed to deposit perovskite thin films or microwires for optoelectronic applications^28^,^29^. In contrast to the deposition of perovskite solutes by doctor-blade coating with which the solvent evaporation is driven by applying high temperatures^28^,^29^,^30^, MASP promotes the perovskite crystallization by accelerating the solvent evaporation through the meniscus effect. The crystallization mechanism of perovskite films crafted by MASP is revealed by in-situ optical microscopy investigation into the crystal growth kinetics. As such, it provides insights into the precise control over the crystal morphology and crystallinity of perovskite films. The produced perovskite films possess preferred crystal orientations and enhanced optoelectronic properties. A high PCE approaching 20% is achieved in the planar perovskite solar cells assembled using the MASP-enabled perovskite films as the photoactive layers.

## Results

### Outward convective flow within meniscus perovskite ink

**Figure 1:**
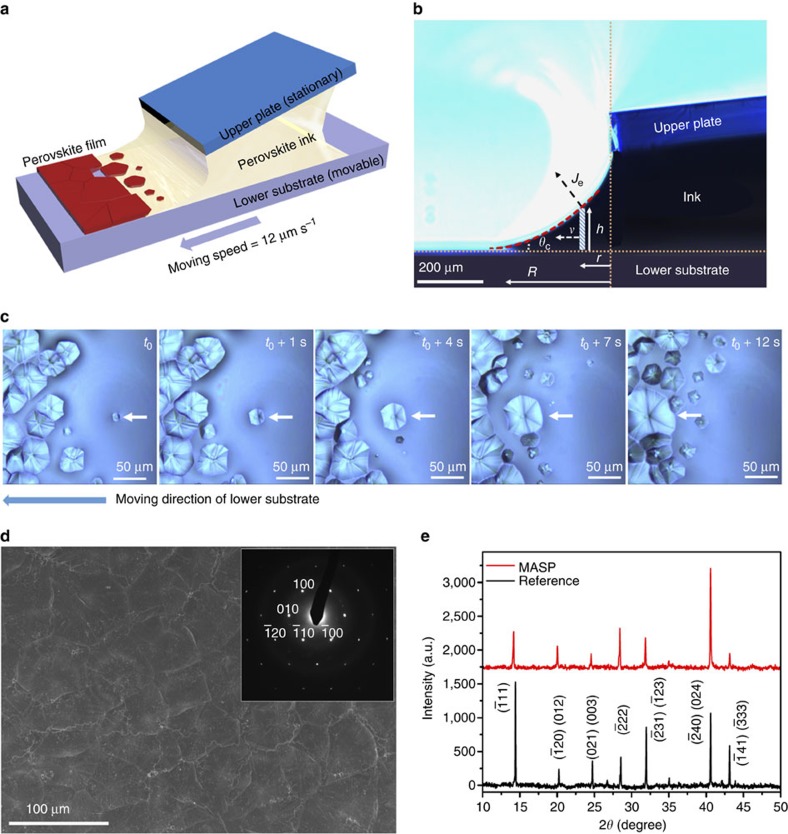
Meniscus-assisted solution printing of FA_0.85_>MA_0.15_PbI_2.55_Br_0.45_ perovskite crystal films. (**a**) Schematic illustration of the meniscus-assisted solution printing (MASP) of large-grained perovskite films. (**b**) Optical micrograph of the side-view meniscus ink confined between a lower flat, movable substrate and an upper stationary plate. (**c**) Optical micrograph of the microstructural evolution of FA_0.85_MA_0.15_PbI_2.55_Br_0.45_ perovskite grain as a function of time. (**d**) Scanning electron microscopy (SEM) image of the perovskite film crafted by MASP. A representative selected area electron diffraction (SAED) of the perovskite film is shown as an inset. (**e**) X-ray diffraction (XRD) profiles of the perovskite film crafted by MASP (upper) and the reference sample prepared by spin coating (lower).

A complex stoichiometry of FA_0.85_MA_0.15_PbI_2.55_Br_0.45_ containing two mixed organic cations (that is, FA_0.85_MA_0.15_) and two mixed halides (that is, I_2.55_Br_0.45_) is selected as the perovskite material because of its enhanced efficiency and stability as compared to MAPbI_3_ (ref. 31). In particular, FAPbI_3_ has a lower bandgap than that of MAPbI_3_ and thus is beneficial for increasing light harvesting, and MAPbBr_3_ improves the stability of the FAPbI_3_ perovskite phase. A scheme of crafting large perovskite crystal grains by MASP is illustrated in Fig. 1a. The metal halide perovskite ink FA_0.85_MA_0.15_PbI_2.55_Br_0.45_ in DMSO was loaded in a two-nearly-parallel-plate geometry where the lower flat substrate can be moved against the upper stationary plate in a programmable manner. The separation distance between these two plates was fixed at 300 μm to form a concave meniscus held by capillary force. An important signature of capillary action is the presence of a meniscus at the edge of the confined ink. Figure 1b shows a representative side-view optical micrograph of confined meniscus ink, where the velocity of outward convective flow *v* induced by solvent evaporation can be described by Navier-Stokes equation with the lubrication approximation (Supplementary Note 1)^22^,^32^:


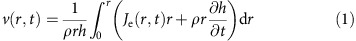


where *J*_e_ is the evaporative flux of solvent, *t* is time, *ρ* is the density of solution, *r* is the horizontal distance of the meniscus surface away from the bulk ink, and *h* is the height of the meniscus at *r*. As the volume of loaded ink is sufficient for completing the printing process, the shape of the meniscus remains relatively unchanged with time, and thus the *∂h/∂t* term can be neglected. Equation (1) can then be simplified as:


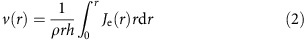


We fitted the experimentally measured outline of the meniscus with a parabolic equation *h*=*a*·(*R*−*r*)^b^, where *h* is dependent on the moving speed of the lower substrate (that is, the coating speed of perovskite). The theoretical fit agreed well the experimental meniscus outline (that is, the red dashed curve in Fig. 1b). The evaporative flux *J*_e_ can be described by the non-equilibrium one-sided (NEOS) model (Supplementary Note 1)^33^:


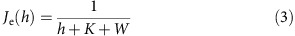


where *K* is the non-equilibrium parameter^34^, *W* is the thermal effect given by 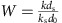 (*k* is the liquid thermal conductivity, *k*_s_ is the thermal conductivity of substrate, *d*_0_ is the liquid thickness and *d*_s_ is the thickness of substrate). By combining Equation (3) with Equation (2), the outward flow velocity *v* as a function of the distance *r* away from the bulk ink can be obtained:


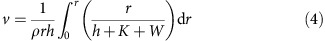


According to Equations (3) and (4), we can derive that the outward convective flow increases sharply at the meniscus edge (Supplementary Fig. 1), as the solvent evaporation is faster toward the edge of the meniscus ink, correspondingly transporting the solutes to the meniscus front and leading to the perovskite supersaturated phase at the edge.

### Process of meniscus-assisted solution printing

As a result, FA_0.85_MA_0.15_PbI_2.55_Br_0.45_ solutes gradually precipitate out from the supersaturated phase and grow into large crystals as the outward flow continuously transport more solutes to the edge of the meniscus. A real-time tracking of the perovskite crystal growth by optical microscopy is shown in Fig. 1c, depicting the evolution of a perovskite nucleus into a large-grained microstructure (Supplementary Movie 1). At time *t*_0_, a relatively small perovskite grain (marked with the white arrow) appeared in the front of meniscus ink with an effective diameter of ∼4 μm. As time progressed (that is, from *t*_0_*+*1 s, *t*_0_*+*4 s, to *t*_0_*+*7 s), the grain underwent a two-dimensional (2D) growth to a large crystal island (Supplementary Fig. 2a). Eventually, the grain growth slowed down when it was connected with other adjacent grains (that is, *t*_0_*+*12 s), accompanied by the coalescence of neighbouring grains (Supplementary Fig. 2b). We note that the perovskite grain growth is expected to initially follow a normal three-dimensional (3D) mode before its size becomes comparable to the film thickness (that is, ∼900 nm, Supplementary Fig. 3)^18^. However, the grain size at the initial stage was too small to be accurately seen and tracked by optical microscopy. Once the grain size exceeded the film thickness, the perpendicular growth was suppressed due to the specimen thickness effect in thin films^35^, resulting in the observed 2D growth.

The lower substrate mounted on the translational stage was moved at a constant speed of 12 μm s^−1^ in the direction opposite to the receding meniscus. As a result, the evaporative front progressively swept across the entire substrate to print a continuous film composed of large perovskite grains (Supplementary Movie 2), and no stick-slip motion was observed. Figure 1d shows a representative scanning electron microscopy (SEM) image of the perovskite film crafted by MASP, revealing that FA_0.85_MA_0.15_PbI_2.55_Br_0.45_ perovskite grains with a size of 20–80 μm were densely packed together without observable voids at grain boundaries. A low-magnification SEM image in Supplementary Fig. 4a further displays a uniform morphology over a few millimetres. We observed that a uniform morphology can only be achieved when the coating speed *v*_c_ (that is, *v*_c_=12 μm s^−1^) was close to the receding speed of the meniscus edge due to solvent evaporation. When the coating speed (for example, *v*_c_=2 μm s^−1^) was slower than the meniscus receding speed, the meniscus edge was found to quickly reach the edge of the upper plate. As a result, the perovskite crystals bridged the upper plate and the lower substrate (Supplementary Fig. 4b). On the other hand, when the coating speed (for example, *v*_c_=50 μm s^−1^) was faster than the meniscus receding speed, a liquid layer rather than a solid film was coated on the substrate. The liquid layer then dried to form discontinuous perovskite crystals as shown in Supplementary Fig. 4c. Recently, a physical model has been built to predict the optimal window for the coating speed by estimating the equilibrium front evaporation speed *v*_efe_ in the evaporation regime of meniscus coating^36^. We employ this model to further analyse the influence of the substrate temperature, the boiling point of solvent, the solvent density, and the meniscus geometrical factor in the MASP process on the front evaporation speed of DMSO (Supplementary Note 2). The calculated *v*_efe_ of DMSO at 60 °C is 4 μm s^−1^ (Supplementary Fig. 5a) and the optimized coating speed *v*_c_ we used in MASP was 12 μm s^−1^, thus the normalized coating speed 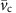
=*v*_c_/*v*_efe_=3, suggesting that this model may be able to predict the optimal window of the coating speed via estimating the front solvent evaporation rate. In addition, we found that there was also an optimal substrate temperature (that is, *T=*60±10 °C) for depositing uniform FA_0.85_MA_0.15_PbI_2.55_Br_0.45_ perovskite films by MASP. Following this optimal operation window (that is, *v*_c_=12 μm s^−1^ and *T*=60 °C), the two-nearly-parallel-plate geometry, the substrate temperature, and the coating speed were thus fixed. We can adjust the film thickness by simply varying the perovskite precursor solution concentration. Supplementary Fig. 5b shows the thickness of MASP-deposited FA_0.85_MA_0.15_PbI_2.55_Br_0.45_ perovskite film as a function of the solution concentration. We also characterized the morphology of the spin-coated FA_0.85_MA_0.15_PbI_2.55_Br_0.45_ perovskite film as reference. The SEM image showed that the spin-coated perovskite film comprised the nanoscale grains with a size of 50–300 nm (Supplementary Fig. 6a). The cross-sectional SEM image revealed a typical film thickness of 780±20 nm (Supplementary Fig. 6b). The elemental mapping of the spin-coated FA_0.85_MA_0.15_PbI_2.55_Br_0.45_ perovskite film was measured by energy-dispersive X-ray spectroscopy (EDS), verifying that the spin-coated film had a uniform composition dispersion (that is, Pb, Br and I) (Supplementary Fig. 7).

The presence of high-quality micrometre-sized grains within these large FA_0.85_MA_0.15_PbI_2.55_Br_0.45_ perovskite grains was corroborated by the well-defined selected area electron diffraction (SAED) pattern (that is, selected area size=2 μm, Supplementary Fig. 8) shown as an inset in Fig. 1d, which can be attributed to a trigonal perovskite crystal with the (110) plane preferentially parallel to the substrate (Supplementary Method 1; Supplementary Fig. 9)^37^. However, the spin-coated FA_0.85_MA_0.15_PbI_2.55_Br_0.45_ perovskite film showed ring-type SAED patterns (Supplementary Fig. 10), implying that there was no preferred crystal orientation and the film had a polycrystalline structure. The X-ray diffraction (XRD) profiles of the FA_0.85_MA_0.15_PbI_2.55_Br_0.45_ perovskite film prepared by MASP and the reference sample prepared by spin coating are shown in Fig. 1e. All diffraction peak positions of these two films agreed well with the simulated XRD profile of a trigonal perovskite crystal with the space group *P*3*m*1. Notably, the relative intensity ratio of (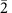
40)/(024) to (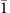
11) was increased for the film prepared by MASP, implying that the MASP film possessed the preferred crystal orientation along the <
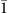
20>/<012> direction (Supplementary Method 2; Supplementary Fig. 11). In particular, the <012>-oriented FA_0.85_MA_0.15_PbI_2.55_Br_0.45_ trigonal crystal forms a corner-sharing lead halide octahedral network with a long-range order (Supplementary Fig. 9d), which is an ideal structure for effective charge transport across the perovskite film^38^. It is noteworthy that the high-purity trigonal perovskite crystals were directly formed at a relatively low temperature (that is, 60 °C) by MASP (Supplementary Fig. 12), thereby dispensing with the need for high-temperature treatment in the spin-coated films in order to convert the DMSO-incorporated intermediate to perovskite phase (Supplementary Note 3). We also note that the diffraction peak positions of the FA_0.85_MA_0.15_PbI_2.55_Br_0.45_ perovskite film prepared by MASP were not completely the same as those prepared by spin coating. For example, there is a small right shift of 2*θ*=0.26^o^ for the (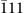) peak in the spin-coated film. The differences in the diffraction peak positions suggested that the stoichiometry of the perovskite films prepared by MASP and spin coating may be slightly different. Thus, X-ray photoelectron spectroscopy (XPS) measurements were performed to identify the chemical composition of perovskite films. Clearly, the perovskite film prepared by MASP had the real chemical composition of (FAPbI_3_)_0.85_(MAPbBr_3_)_0.14_ (Supplementary Fig. 13), which was almost consistent with the stoichiometry of the perovskite precursors in the ink. However, for the spin-coated perovskite film, the real chemical composition was found to be (FAPbI_3_)_0.82_(MAPbBr_3_)_0.13._ The MASP technique not only entail a relatively low-temperature deposition of FA_0.85_MA_0.15_PbI_2.55_Br_0.45_ perovskite films with high crystallinity, but also facilitate the formation of micrometer-scale perovskite grains with preferred crystal orientations. Conceivably, the resulting perovskite films may demonstrate advantageous optoelectronic properties.

### Growth kinetics of perovskite crystals

**Figure 2:**
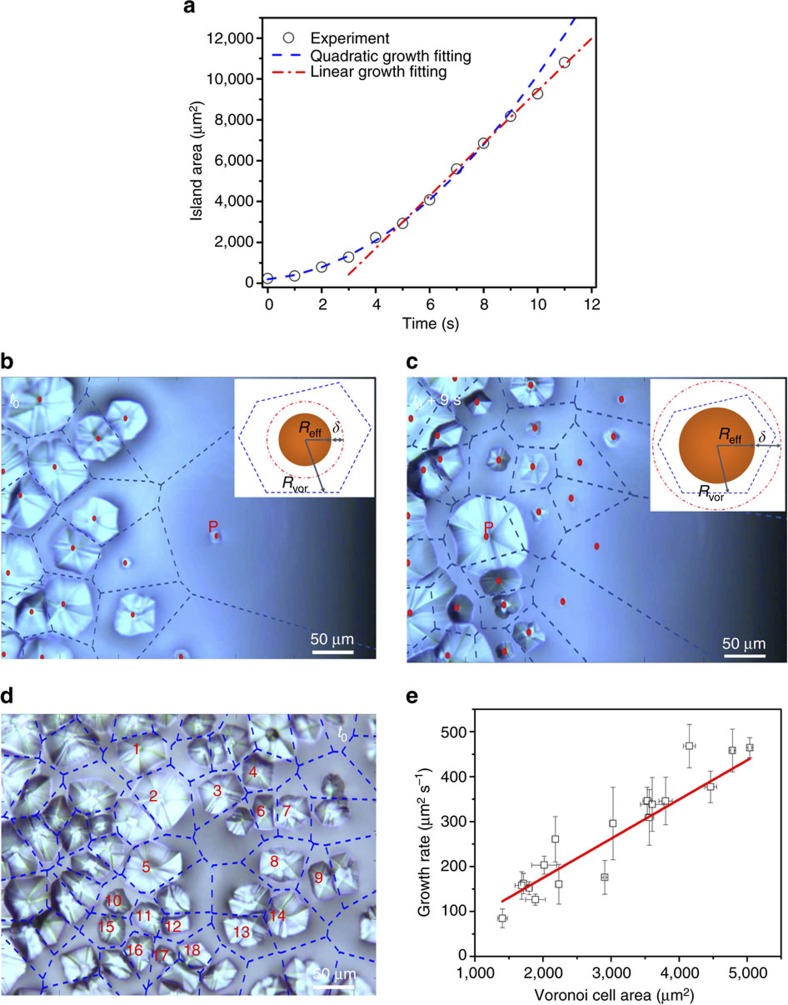
Growth kinetics of FA_0.85_MA_0.15_PbI_2.55_Br_0.45_ perovskite crystals during meniscus-assisted solution printing. (**a**) Plot of the area of the tracked FA_0.85_MA_0.15_PbI_2.55_Br_0.45_ perovskite crystal island as a function of time. The tracked island centre is marked with P in **b** and **c**. (**b**) Voronoi cell diagram of perovskite islands at time *t*_0_, and the centre of the tracked island is labelled as P. The inset illustrates the topological relationship of *R*_eff_+*δ*<*R*_Vor_, where *R*_eff_ is the effective radius of the perovskite island, *δ* is the solute diffusion length, and *R*_Vor_ is the effective radius of the corresponding Voronoi cell. (**c**) Voronoi cell diagram of perovskite islands at time *t*_0_*+*9 s, and the centre of the tracked island is labelled as P. The inset depicts the topological relationship of *R*_eff_+*δ*>*R*_Vor_. (**d**) Eighteen selected perovskite islands and the corresponding Voronoi cells for tracking the perovskite crystal growth rate as a function of the Voronoi cell area for each crystal island from time 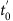 to 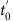
*+*3 s. (**e**) Plot of the average growth rate of the eighteen perovskite crystal islands as a function of their Voronoi cell areas. Error bars denote the s.d. of the data collected from time 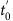 to 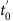
*+*3 s.

To further understand the crystallization mechanism of perovskites crafted by MASP, we turn our attention to scrutinize the growth kinetics of FA_0.85_MA_0.15_PbI_2.55_Br_0.45_ crystals by real-time optical microscopy measurement. Figure 2a depicts the area of the tracked FA_0.85_MA_0.15_PbI_2.55_Br_0.45_ crystal island as a function of time. A two-stage crystal growth is observed, namely, a quadratic increase of crystal area with time at the early stage of growth and a linear increase of crystal area with time at the late stage. It is worth noting that this growth pattern is analogous to the vacuum deposition of organic crystals on substrate^39^,^40^, except that the atom transferring medium in MASP is a liquid flow. In this context, we examine the perovskite crystal growth kinetics according to the scaling capture zone model^41^,^42^, in which the crystal growth is essentially governed by the accessible collection area for solutes and the solute diffusion length^41^. The accessible collection area of the FA_0.85_MA_0.15_PbI_2.55_Br_0.45_ crystal island can be estimated by its Voronoi cell that included all solutes closer to this specific island than to any others^43^. The central point of each island (that is, red dots in Fig. 2b,c) is labelled as the nucleation centre to diagram the Voronoi cell. The good agreement between the Voronoi cell topographies and the obtained perovskite island shapes suggests a strong correlation between the crystal growth and the accessible collection area^40^. At the early growth stage of the FA_0.85_MA_0.15_PbI_2.55_Br_0.45_ perovskite island with its centre labelled as P (Fig. 2b), the effective radius *R*_eff_ of the island was relatively small (that is, *R*_eff,t0_ =∼4 μm at time *t*_0_), the Voronoi cell radius *R*_Vor_ of the island at time *t*_0_ was very large (that is, *R*_Vor,t0_>100 μm) as it was isolated far from other neighbouring crystals. In the case of *R*_eff_+*δ*<*R*_Vor_, the crystal growth rate was mainly determined by the solute diffusion length *δ* toward the island, and thus a quadratic crystal growth emerged and can be described by d*A/*d*t=k*_q_(2π*δR*_eff_*+πδ*^2^), where *A=π*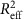 is the island area, and *k*_q_ is the rate constant^39^,^44^. The value of *δ* was then derived by fitting the measured island size to the quadratic growth equation, yielding *δ*=4 μm. We note that the EDS measurement showed a uniform elemental mapping of the FA_0.85_MA_0.15_PbI_2.55_Br_0.45_ perovskite island (Supplementary Fig. 14), suggesting that the diffusion lengths of various solutes (that is, FA, MA, Pb, I and Br) were possibly comparable and did not generate obvious composition segregation in perovskite crystals during MASP. The evaporation-induced outward convective flow is expected to facilitate the solute diffusion and promote the crystal growth rate at this stage. The relationship *R*_eff_+*δ*<*R*_Vor_ changed to *R*_eff_+*δ*>*R*_Vor,_ as the FA_0.85_MA_0.15_PbI_2.55_Br_0.45_ perovskite island grew larger (that is, *R*_eff,t9_=∼51 μm at time *t*_0_*+*9 s). Meanwhile more new adjacent crystals emerged as evidenced in Fig. 2c, the tracked island had to compete with the neighbours for collecting the perovskite solutes, leading to smaller *R*_Vor_ (that is, *R*_Vor,t9_=∼48 μm at time *t*_0_*+*9 s). As a result, the crystal growth rate was largely determined by the accessible collection area of the island, giving rise to a linear crystal growth (d*A/*d*t*=*k*_l_*A*_Vor_, where *A*_Vor_ is the area of the Voronoi cell, and *k*_l_ is the rate constant)^39^,^44^.

We also investigate the crystal growth rates of FA_1−*x*_MA_*x*_PbI_3(1−*x*)_Br_3*x*_ perovskites with varied chemical compositions (0.05≤*x*≤0.25), and they all follow the two-stage growth kinetics (Supplementary Fig. 15). In order to further verify the reliability of the observed crystal growth kinetics, we analyse the growth rates of a number of FA_0.85_MA_0.15_PbI_2.55_Br_0.45_ crystal islands (that is, a total of 18 islands shown in Fig. 2d) in a single real-time tracking by optical microscopy. As the islands were isolated far from each other at the early stage, only the growth rates at the late stage were monitored, that is, the crystal growth followed the condition of *R*_eff_ + *δ*>*R*_Vor_. The average crystal growth rate and the corresponding Voronoi cell area of each island from time 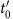 to 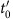
*+*3 s were calculated (Supplementary Fig. 16; Supplementary Movie 3). A linear relationship d*A/*d*t=k*_l_*A*_Vor_ was indeed also observed for these islands with varied sizes and growth rates (Fig. 2e). The origin of the linear crystal growth is that the solute density varies slowly in the crystallization regime when the solute diffusion rate is comparable with the solute crystallization rate, and the crystallization regime can be approximated as a steady state, thereby enabling the statistics studies of the diffusion equation with appropriate boundary conditions for simple island configuration^42^. On the basis of this crystal growth kinetics, it is suggested that the final size of the perovskite crystals yielded by MASP depends strongly on the nucleation density, and a lower nucleation density facilitates the growth of large crystals due to a fewer competitors for solutes. Furthermore, the nucleation density relies on the evaporation-driven outward convective flow (that is, faster outward flow leading to higher nucleation density) within the meniscus ink, which can be tuned by adjusting the meniscus height, the solvent density, the substrate temperature, and the substrate moving speed^36^,^45^.

### Optoelectronic performances of perovskites films

**Figure 3:**
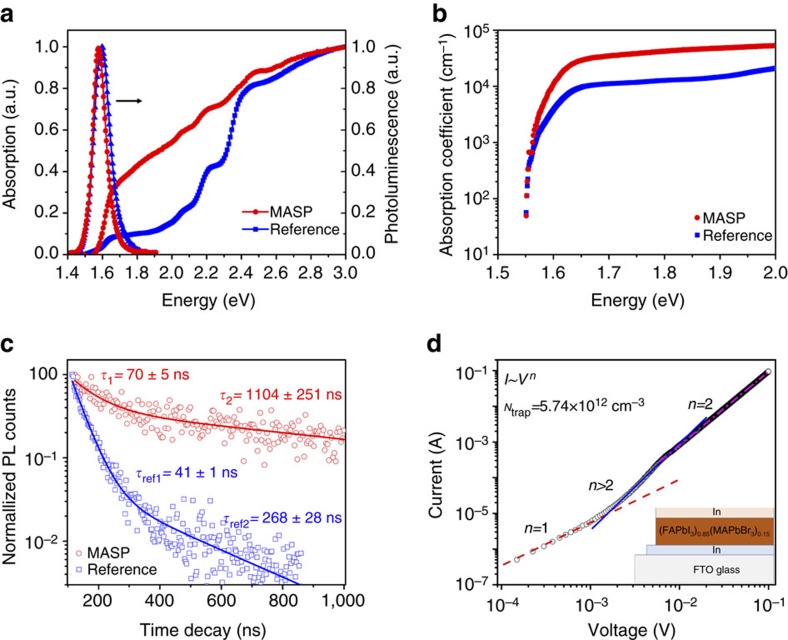
Optoelectronic properties of FA_0.85_MA_0.15_PbI_2.55_Br_0.45_ perovskite films crafted by meniscus-assisted solution printing. (**a**) Ultraviolet–visible (UV–vis) absorption and photoluminescence (PL) spectra of the FA_0.85_MA_0.15_PbI_2.55_Br_0.45_ perovskite film crafted by meniscus-assisted solution printing (MASP) and the reference sample prepared by spin coating. (**b**) Urbach tails of the perovskite films prepared by MASP (solid circles) and spin coating (squares), respectively. (**c**) Time-resolved photoluminescence (TRPL) decay traces of the perovskite films prepared by MASP (open circles) and spin coating (open squares), respectively. (**d**) Current–voltage curve of the perovskite film crafted by MASP for space charge limited current (SCLC) analysis. The inset depicts the SCLC architecture.

Intrigued by the attractive features of micrometer-sized grains and preferred crystal orientations, we set out to investigate the photophysical properties of MASP-enabled perovskite films. Figure 3a compares the ultraviolet-visible (UV–Vis) absorption and photoluminescence (PL) spectra of the FA_0.85_MA_0.15_PbI_2.55_Br_0.45_ perovskite film crafted by MASP and the film prepared by spin coating (Supplementary Method 3). The PL peak of the MASP film exhibited a ∼20 meV red-shift compared to that of the spin-coated film (Supplementary Fig. 17a). Since the Stokes shifts for both films were almost negligible (that is, ∼5 meV), accordingly, a red-shift of the absorption onset edge was seen (Supplementary Fig. 17b and 17c), implying that the MASP film had a slightly lower bandgap than that of the spin-coated film. Moreover, the full width at half maximum (FWHM) of the PL spectrum was narrowed by ∼26 meV for the MASP film, suggesting that the MASP film contained fewer crystalline defects^16^,^46^. The low Urbach energy (that is, ∼19 meV, Fig. 3b) further confirmed the presence of reduced localization disordering within the MASP-enabled FA_0.85_MA_0.15_PbI_2.55_Br_0.45_ perovskite film^47^. The charge carrier lifetime was measured by time-resolved photoluminescence (TRPL) spectroscopy (Fig. 3c). The charge carrier lifetimes in the MASP perovskite film (that is, *τ*_1_=70±5 ns and *τ*_2_=1,104±251 ns) were much longer than the spin-coated film (that is, *τ*_ref1_=41±1 ns and *τ*_ref2_=268±28 ns), indicating a lower trap density within the MASP film. Particularly, the longer lifetime *τ*_2_ of the MASP film exceeded 1,000 ns, signifying that the trap-induced carrier recombination was appreciably reduced due to the formation of high-quality and large-sized FA_0.85_MA_0.15_PbI_2.55_Br_0.45_ perovskite grains by MASP^48^. The charge trap densities within the perovskite films were actually measured from the space charge-limited current (SCLC) devices (Supplementary Method 4)^46^. The charge trap density within the MASP film was estimated to be ∼5.74 × 10^12^ cm^−3^ (Fig. 3d), which was much lower than that of the spin-coated film (that is, ∼4.04 × 10^15^ cm^−3^; Supplementary Fig. 18), in good agreement with the UV–Vis and PL spectroscopy studies. Taken together, the results noted above substantiate that the MASP method enable the formation of FA_0.85_MA_0.15_PbI_2.55_Br_0.45_ perovskite film with good crystallization and low charge traps, offering the remarkable potential to yield high-performance optoelectronic devices.

**Figure 4:**
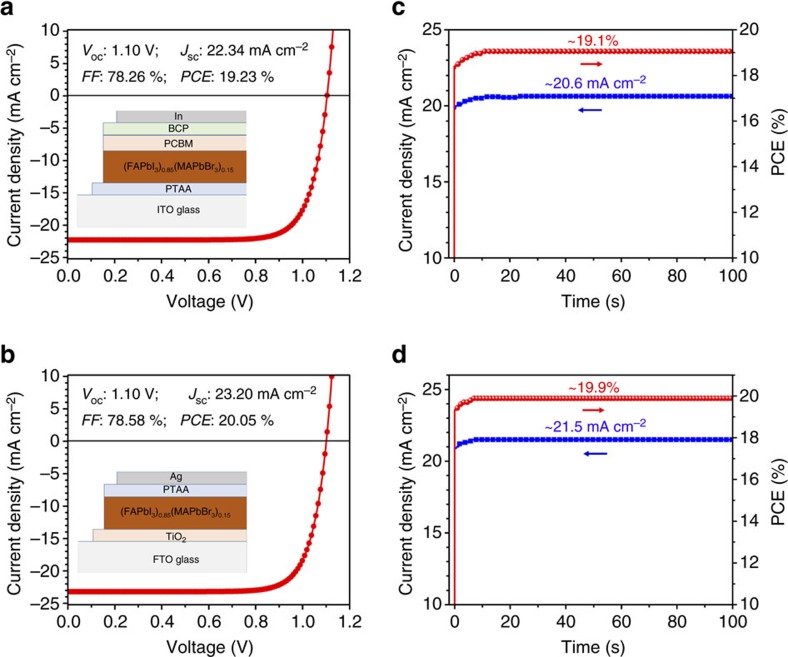
Photovoltaic performances of FA_0.85_MA_0.15_PbI_2.55_Br_0.45_ perovskite solar cells. (**a**) Current density–voltage (*J*–*V*) characteristic of the inverted planar FA_0.85_MA_0.15_PbI_2.55_Br_0.45_ perovskite solar cell. The device architecture is shown as an inset. (**b**) *J*–*V* characteristic of the standard planar perovskite solar cell. The device architecture is shown as an inset. (**c**) Photocurrent density and the corresponding efficiency of the inverted planar perovskite solar cell measured as a function time at a constant forward bias of 0.925 V under an AM1.5G standard sunlight. (**d**) Photocurrent density and the corresponding efficiency of the standard planar perovskite solar cell measured as a function time at a constant forward bias of 0.925 V under an AM1.5G standard sunlight.

We thus fabricate FA_0.85_MA_0.15_PbI_2.55_Br_0.45_ perovskite solar cells via MASP, which allows for direct deposition of a perovskite photoactive layer on the top of the functional electrodes. The effect of perovskite film thickness on the photovoltaic performance is investigated. The film thickness of the MASP perovskite film is varied by adjusting the ink concentration (Supplementary Fig. 5b). The corresponding photovoltaic efficiency with the device architecture of FTO/compact-TiO_2_/Perovskite/PTAA/Ag is studied. Supplementary Fig. 19 compares the current density–voltage (*J*–*V*) curves of the devices with the perovskite film thickness ranging from ∼600 nm to ∼1,400 nm. The photovoltaic parameters are summarized in Supplementary Table 1. When the film thickness was increased from 620 to 890 nm, the *J*_sc_ and *FF* were increased, resulting in the increase in *PCE* from 15.73 to 18.27%. When the film thickness was further increased to 1,070 nm, the *FF* tended to decrease. Notably, all photovoltaic parameters decreased when the film thickness reached 1,410 nm, probably due to the increased series resistance and charge recombination in the thick film. Thus, the optimal film thickness was fixed at ∼900 nm in this work. In this context, the inverted planar solar cells with the device architecture of ITO/PTAA/Perovskite/PCBM/BCP/Ag produced an average PCE of 18.49±0.47% (Supplementary Table 2). The champion cell achieved a PCE of 19.23% with an open-circuit voltage *V*_oc_ of 1.10 V, short-circuit current *J*_sc_ of 22.34 mA cm^−2^, and fill factor FF of 78.26% (Fig. 4a). A stable output photocurrent density of 20.6 mA cm^−2^ and PCE of 19.1% of this champion cell were obtained under the standard sunlight (Fig. 4c). Compared to inverted planar devices, higher PCEs were achieved in the standard planar devices with the architecture of FTO/compact-TiO_2_/Perovskite/PTAA/Ag, in which the average PCE was 19.22±0.36% (Supplementary Table 3). The champion cell delivered a *V*_oc_ of 1.10 V, *J*_sc_ of 23.20 mA cm^−2^, FF of 78.58% and a PCE of 20.05% (Fig. 4b). Similarly, the stable output photocurrent density and PCE of the champion cell reached 21.5 mA cm^−2^ and 19.9% (Fig. 4d). The integrated current densities from the external quantum efficiency (EQE) measurements were 21.94 and 22.38 mA cm^−2^ for champion inverted and standard planar devices (Supplementary Fig. 20), respectively, which agreed well with the measured *J*_sc_ values. Compared with those of spin-coated perovskite films (Supplementary Tables 4 and 5), the MASP-enabled standard and inverted planar perovskite solar cells displayed not only high efficiencies with inappreciable hysteresis (Supplementary Fig. 21) but also small s.d. in the average PCEs. This can be attributed to the presence of large-sized, highly-crystallized and preferentially-oriented perovskite grains created by MASP.

## Discussion

**Figure 5:**
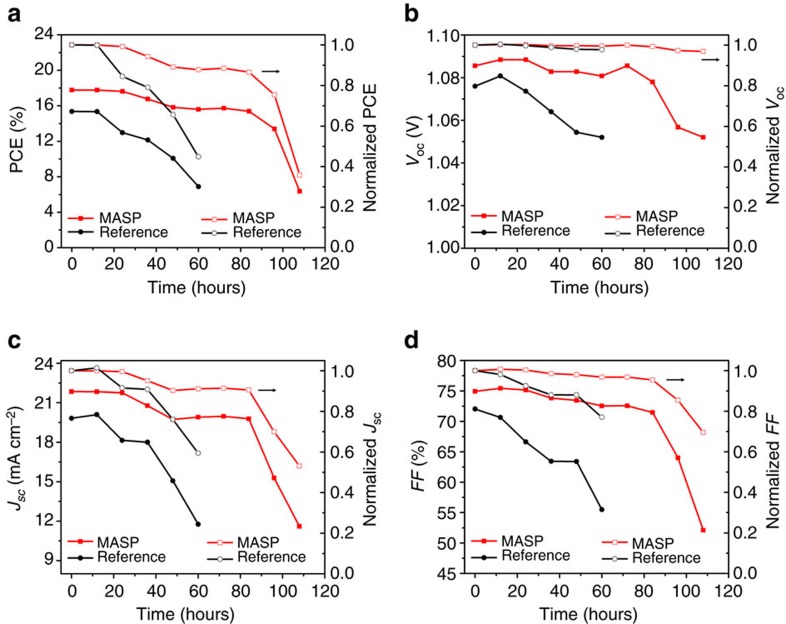
Device stability under continuous working condition. Stability plots of (**a**) PCE, (**b**) *V*_oc_, (**c**) *J*_sc_ and (**d**) FF as a function of time (left axis). The normalized values are also included for better comparisons (right axis). The device stability was measured under continuous AM1.5G solar illumination in ambient environment without encapsulation.

The stability of FA_0.85_MA_0.15_PbI_2.55_Br_0.45_ perovskite solar cells with the device architecture of FTO/compact-TiO_2_/Perovskite/PTAA/Ag fabricated by MASP and spin coating, respectively, is also investigated. The devices are continuously exposed to AM1.5G solar illumination in ambient environment (temperature=25±2 °C and relative humidity=30±8%) without encapsulation. The photovoltaic efficiency is measured at an interval of 12 h. Figure 5 compares the photovoltaic parameters as a function of time for both MASP-deposited and spin-coated devices (the *J*–*V* curves were shown in Supplementary Fig. 22). The spin-coated device started to experience the efficiency drop after 12-h continuous illumination and lost 50% of its initial efficiency in 60 h. In contrast, the MASP-deposited device maintained nearly 90% of its initial efficiency after 60-h continuous illumination in air. The efficiency was then rapidly decreased after 84 h and reached 35% of its initial efficiency in 108 h. The enhanced stability of perovskite films prepared by MASP can be ascribed to the incorporation of FA^+^ and Br^-^ components which are beneficial for improving the perovskite stability^31^, as the unsealed CH_3_NH_3_PbI_3_ perovskite solar cells usually lose 70% of their initial efficiencies within 48 h under continuous illumination in air^49^,^50^. Moreover, it is also highly correlated with the high-quality large perovskite grains produced by MASP which are more resistant to light- and moisture-induced degradation. Our ongoing work on further improving the device stability involves the incorporation of Cs^+^ in the perovskite composition^51^ and the encapsulation of devices to isolate them from moisture and UV light. These results will be reported elsewhere in the future.

We also explore the manufacturing of large-area perovskite solar cells by MASP. The large-area device is fabricated with the architecture of FTO/compact-TiO_2_/perovskite/PTAA/Ag and the active-layer area of 1.00±0.02 cm^2^. A showdow mask with the aperture area of 0.980 cm^2^ is used to measure the *J*–*V* curves. The highest PCE of 18.02% in a backward scanning was obtained (Supplementary Fig. 23). The hysteresis phenomenon was obvious compared to the standard-area devices (that is, photoactive area=0.10 cm^2^). However, it is notable that the MASP process is more complicated for depositing large-area perovskite films, as the crystallization rate tends to change and become unstable at the late printing stage. Such instability is likely to be caued by the change of the mensicus shape during the MASP process as the perovskite ink is consumed. Thus, the MASP apparatus will need to be modified to enable a continuous supply of perovksite ink for maintaining the meniscus shape for large-area printing. Moreover, the deposition of uniform electron-transport layer and hole-trasnport layer by MASP over large areas is also important in further improving the device efficiency.

In summary, we develop a robust and convenient MASP strategy to effectively deposit large-grained dense FA_0.85_MA_0.15_PbI_2.55_Br_0.45_ perovskite films with enhanced optoelectronic properties for high-efficiency perovskite solar cells. Central to this strategy is the solvent evaporation-triggered outward convective flow that transported the perovskite solutes to the edge of the meniscus, promoting the formation of solution-printed micrometre-scale perovskite grains with preferred crystal orientations at low temperatures. The insight into the crystal growth kinetics of FA_0.85_MA_0.15_PbI_2.55_Br_0.45_ perovskite is revealed through the real-time optical microscopy study, from which a two-stage, namely, quadratical followed by linear growth of perovskite crystallization is identified. High photovoltaic PCEs approaching 20% are achieved in planar FA_0.85_MA_0.15_PbI_2.55_Br_0.45_ perovskite solar cells, which can be ascribed to low trap-state density and long carrier lifetimes in large-grained perovskite films crafted by MASP. As the optoelectronic properties of perovskite films are closely related to film quality, the ability of the MASP strategy to yield dense and uniform films may open up new avenues for low-cost production of large-area flexible solution-printed perovskite-based materials and devices.

## Methods

### Materials

Formamidinium iodide (H_2_N=CHNH_2_I; FAI) and methylammonium bromide (CH_3_NH_3_Br; MABr) were synthesized according to the reported methods^31^, and dried in a vacuum oven at 50 °C for 48 h. Lead iodide (PbI_2_, 99.999%), lead bromide (PbBr_2_, 99.999%), dimethyl sulfoxide (DMSO, ≥99.9%) and other materials were purchased from Sigma-Aldrich and used as received.

### Meniscus-assisted solution printing of perovskite films

The perovskite precursor ink was prepared by dissolving FAI, MABr, PbI_2_ and PbBr_2_ (molar ratio of FAI:MABr:PbI_2_:PbBr_2=_0.85:0.15:2.55:0.45) in DMSO, and stirred at 60 °C for 2 h to form 0.25 M FA_0.85_MA_0.15_PbI_2.55_Br_0.45_ precursor ink. A 20 μl of the ink solution was loaded in a confined geometry composed of two nearly parallel plates (an upper stationary plate of 0.5 cm × 1 cm and a lower movable substrate of 1.5 cm × 2.5 cm; the separation distance between these two plates was 300 μm). The lower flat substrate was mounted on a computer-programed translational stage (Parker Hannifin Corp, Mode MX80LVixBL2b) with a resolution of 100 nm. The lower substrate was heated at ∼60 °C on a Linkam Scientific Precision Temperature Controlled Microscope Stage (Linkam TMS 94 LTS 350) to expedite the evaporation of DMSO. The lower flat substrate placed on the translational stage was moved at a speed of 12 μm s^−1^ in the direction opposite to the receding meniscus. Thus, the evaporative front progressively swept across the entire substrate to print a continuous large-grained perovskite film.

### Spin coating of perovskite films

The perovskite precursor solution was prepared by dissolving FAI, MABr, PbI_2_ and PbBr_2_ (molar ratio of FAI:MABr:PbI_2_:PbBr_2_=0.85:0.15:2.55:0.45) in DMSO. The solution concentration was 1.2 M. The precursor solution was maintained at 90 °C. It was then spin coated on a pre-heated substrate at 1,000 r.p.m. for 5 s and 4,000 r.p.m. for 30 s, followed by thermal annealing at 150 °C for 20 min to form the perovskite film.

### Fabrication of standard planar perovskite solar cells

The FTO glass substrate was treated by oxygen plasma for 10 min, followed by spin coating 0.15 M titanium diisopropoxide bis(acetylacetonate) solution at 2,000 r.p.m. for 30 s. The titanium-containing precursor film was dried at 125 °C for 10 min and sintered at 500 °C for 30 min. The resulting TiO_2_ layer was then immersed in 20 mM TiCl_4_ solution at 65 °C for 20 min, followed by sintering at 550 °C for 30 min to produce a compact TiO_2_ layer on the FTO glass substrate. FA_0.85_MA_0.15_PbI_2.55_Br_0.45_ perovskite film was then deposited on the compact-TiO_2_/FTO substrate by MASP in ambient air at ∼30% relative humidity. 2 ml poly(bis(4-phenyl)(2,4,6-trimethylphenyl)amine) (PTAA) toluene solution (25 mg ml^−1^) was mixed with 29 μl Li-bis(trifluoromethanesulfonyl) imide (Li-TFSI)/acetonitrile (170 mg ml^−1^) and 20 μl 4-tert-butylpyridine (TBP). The resulting PTAA solution was spin coated on the perovskite layer at 5,000 r.p.m. for 30 s. Finally, a 200-nm silver layer was thermally evaporated on the PTAA layer.

### Fabrication of inverted planar perovskite solar cells

10 mg ml^−1^ PTAA toluene solution containing the additives of Li-TFSI and TBP was spin coated on the FTO glass substrate at 2,000 r.p.m. for 60 s, followed by thermal annealing at 100 °C for 5 min. FA_0.85_MA_0.15_PbI_2.55_Br_0.45_ perovskite film was then deposited on the PTAA/FTO substrate by MASP. 50 mg ml^−1^ [6,6]-Phenyl C_61_ butyric acid methyl ester (PCBM) 1,2-dichlorobenzene solution was spin coated on the perovskite layer at 3,000 r.p.m. for 30 s, followed by storing in a covered Petri dish for 6 h. Finally, a 5-nm bathocuproine (BCP) layer and a 200-nm silver layer were sequentially deposited by thermal evaporation.

### Fabrication of space charge limited current devices

The FTO glass substrate was treated by oxygen plasma for 10 min, followed by thermally depositing a 500-nm indium layer. FA_0.85_MA_0.15_PbI_2.55_Br_0.45_ perovskite film was deposited on the indium/FTO substrate by MASP. Another 500-nm indium layer was then thermally deposited on the perovskite layer.

### Fabrication of reference devices

For comparison, all reference devices (that is, standard and inverted planar perovskite solar cells) were fabricated in similar ways, except that the perovskite films were prepared by spin coating.

### Characterizations

The film morphology and elemental mapping of perovskite films were characterized using a LEO 1550 scanning electron microscopy (SEM). Selected area electron diffraction (SAED) was performed on a JEOL 100CX transmission electron microscopy (TEM). X-ray diffraction (XRD) profiles were collected by a PANalytical X’Pert PRO X-ray diffractometer using Cu K*α*_*1*_ radiation (*λ*=1.541 Å) operating at 40 kV and 40 mA. UV-Vis spectra were obtained using a Shimadzu UV2600 spectrophotometer with a photometric integrating sphere. Fluorescence spectra were recorded using a PerkinElmer LS 45 Fluorescence Spectrometer. The *in situ* optical microscopy studies were conducted using a Leica DFC450 C digital microscope camera equipped with a 5 Megapixel CCD sensor. Time-resolved photoluminescence measurements were performed using a Photon Technology International (PTI) LaserStrobe spectrofluorometer equipped with a PTI GL-3300 nanosecond nitrogen laser (*λ*=337 nm) operating at 11 Hz. X-ray photoelectron spectroscopy (XPS) was measured by Thermo K-Alpha XPS system. Perovskite solar cells were tested under AM1.5G irradiation using a Newport LCS-100 Solar Simulator (100 mW cm^−2^, calibrated with a Newport 91150V Reference Cell System). The current density–voltage (*J*–*V*) curves were measured using a Keithley 2601A multisource meter. No preconditioning protocol was used before the characterization. The *J*–*V* curves were measured in air (that is, temperature: 25±2 °C, relative humidity: 30±8 %) without encapsulation. The *J*–*V* curves were scanned in the forward direction unless specified, and were processed at the rate of 50 mV s^−1^ with a sweep delay time of 50 ms. For standard-area solar cells, the active-layer area was 0.10±0.02 cm^2^, and a shadow mask with an aperture area of 0.096 cm^2^ was used to define the measured area. For large-area solar cells, the active-layer area was 1.00±0.02 cm^2^ and the aperture area of the shadow mask was 0.980 cm^2^. The device stability with the active-layer area of 0.10±0.02 cm^2^ and the aperture area of 0.096 cm^2^ was measured under continuous AM1.5G solar illumination in ambient environment (that is, temperature=25±2 °C and relative humidity=30±8%) without encapsulation. The photovoltaic efficiency was measured in an interval of 12 h. The external quantum efficiency (EQE) measurement was performed using a Newport Quantum Efficiency Measurement Kit.

### Data availability

The data that support the findings of this study are available from the corresponding author upon request.

## Additional information

**How to cite this article:** He, M. *et al*. Meniscus-assisted solution printing of large-grained perovskite films for high-efficiency solar cells. *Nat. Commun.* **8,** 16045 doi: 10.1038/ncomms16045 (2017).

**Publisher’s note:** Springer Nature remains neutral with regard to jurisdictional claims in published maps and institutional affiliations.

## Supplementary Material

Supplementary Information

Supplementary Movie 1

Supplementary Movie 2

Supplementary Movie 3
