# Supplementary material for: Meniscus-assisted solution printing of large-grained perovskite films for high-efficiency solar cells
Source: Nat Commun. 2017 Jul 7;8:16045. doi: 10.1038/ncomms16045 (PMC5504348; doi:10.1038/ncomms16045)
Supplement: Supplementary Information [file ncomms16045-s1.pdf]

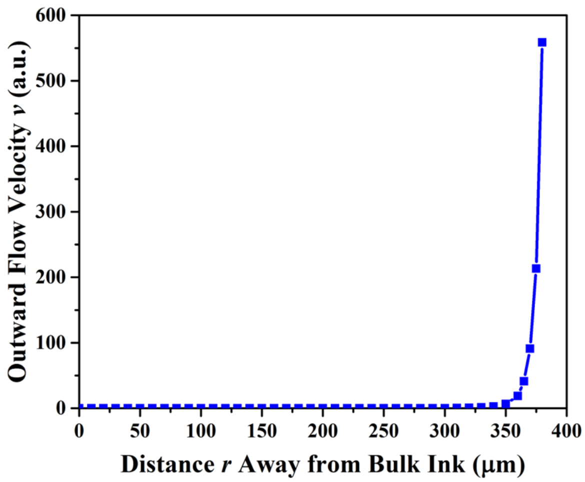


**Supplementary Figure 1 | Plot of the velocity *v* of the outward convective flow as a function of the distance *r* of the meniscus surface away from the bulk ink.**

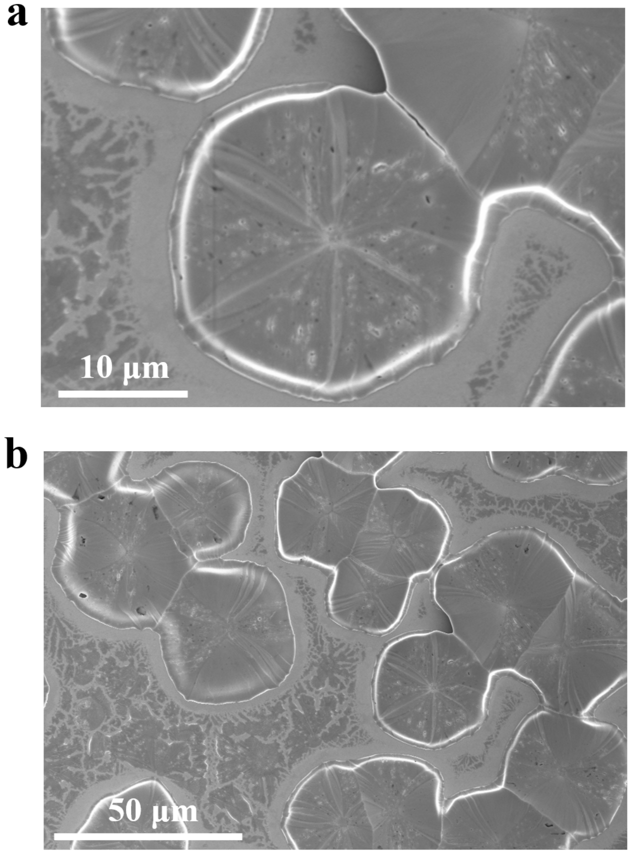


**Supplementary Figure 2 | Scanning electron microscopy images of the $FA_{0.85}MA_{0.15}PbI_{2.55}Br_{0.45}$ perovskite grains formed during the MASP deposition.** (**a**) The morphology of perovskite grain prior to its coalescence with the neighboring grains. (**b**) The coalescence of grain boundaries.

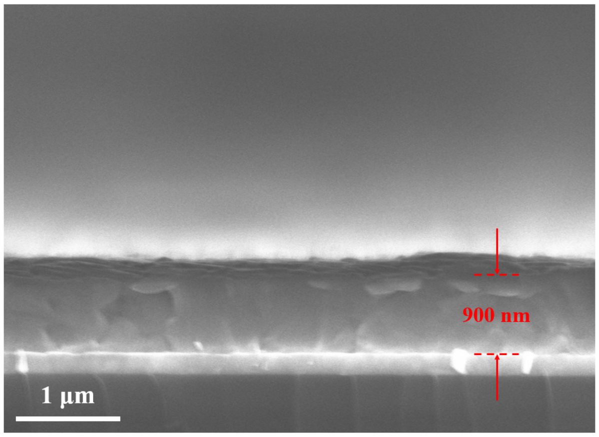


**Supplementary Figure 3 | A side-view scanning electron microscopy image of the MASP-enabled $FA_{0.85}MA_{0.15}PbI_{2.55}Br_{0.45}$ perovskite film with a thickness of ~900 nm.**

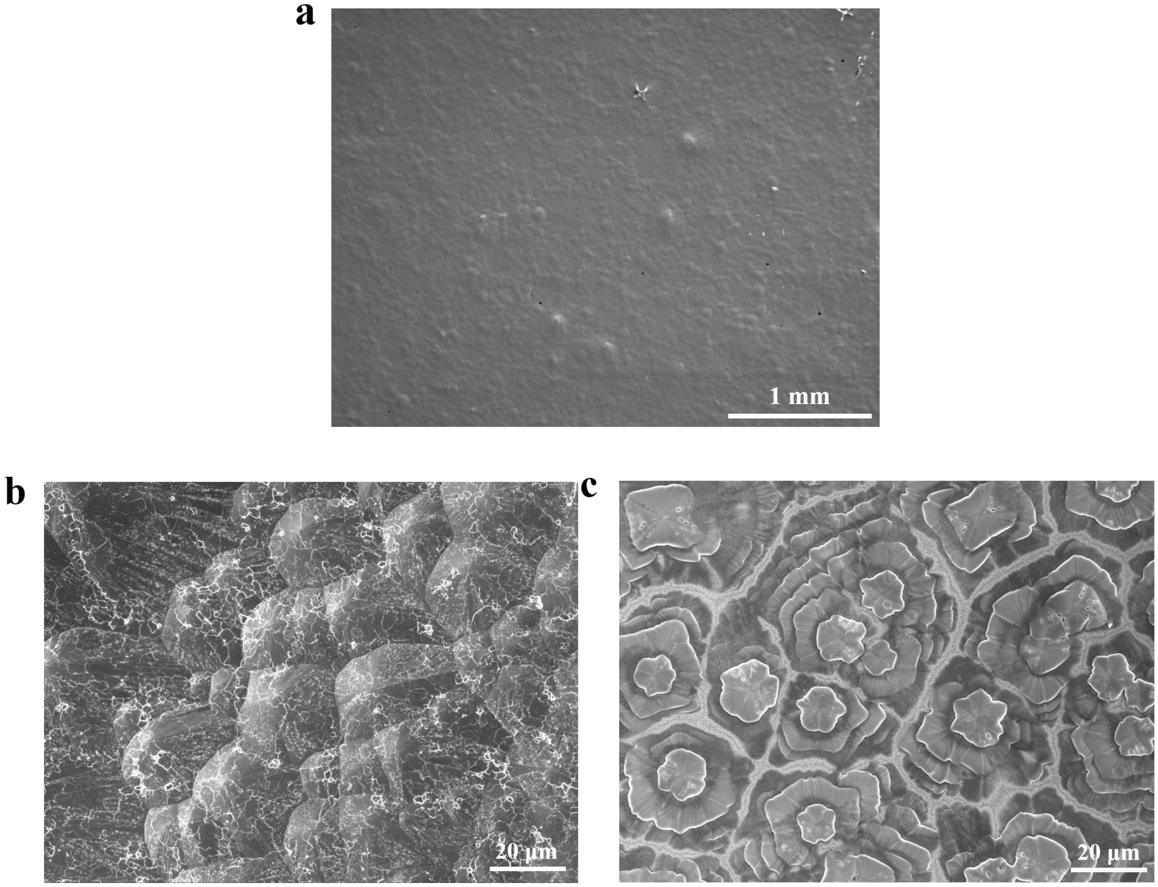


**Supplementary Figure 4 | Scanning electron microscopy images of the $FA_{0.85}MA_{0.15}PbI_{2.55}Br_{0.45}$ perovskite films formed at varied coating speeds.** (**a**) A low-magnification image of the MASP perovskite film. (**b**) The morphology of perovskite film produced at a slow coating speed of 2 μm $s^{-1}$. (**c**) The morphology of perovskite film produced at a fast coating speed of 50 μm $s^{-1}$.

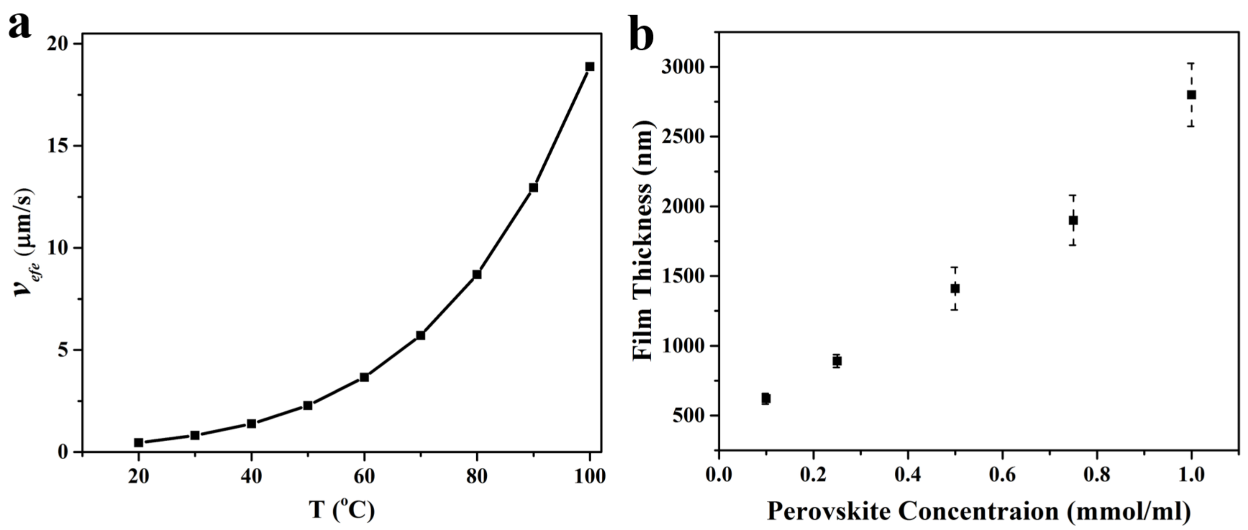


**Supplementary Figure 5 | Effects of temperature and solution concentration on meniscus-assisted solution printing.** (**a**) The equilibrium front evaporation speed $v_{efe}$ of DMSO as a function of temperature $T$ calculated from Supplementary Equation 7. (**b**) The thickness of $FA_{0.85}MA_{0.15}PbI_{2.55}Br_{0.45}$ perovskite film as a function of the concentration of perovskite precursor ink. Error bars denote the standard deviation of the film thicknesses measured from 10 samples for each concentration.

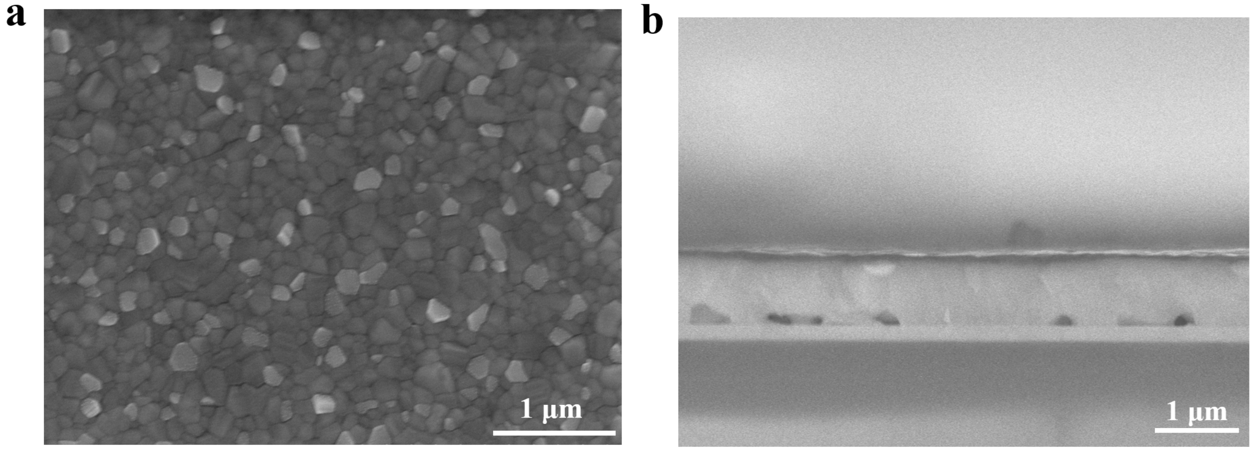


**Supplementary Figure 6 | Scanning electron microscopy images of the $FA_{0.85}MA_{0.15}PbI_{2.55}Br_{0.45}$ perovskite film produced by spin coating.** (**a**) Top view with a size of 50~300 nm. (**b**) Side-view with a thickness of ~780 nm.

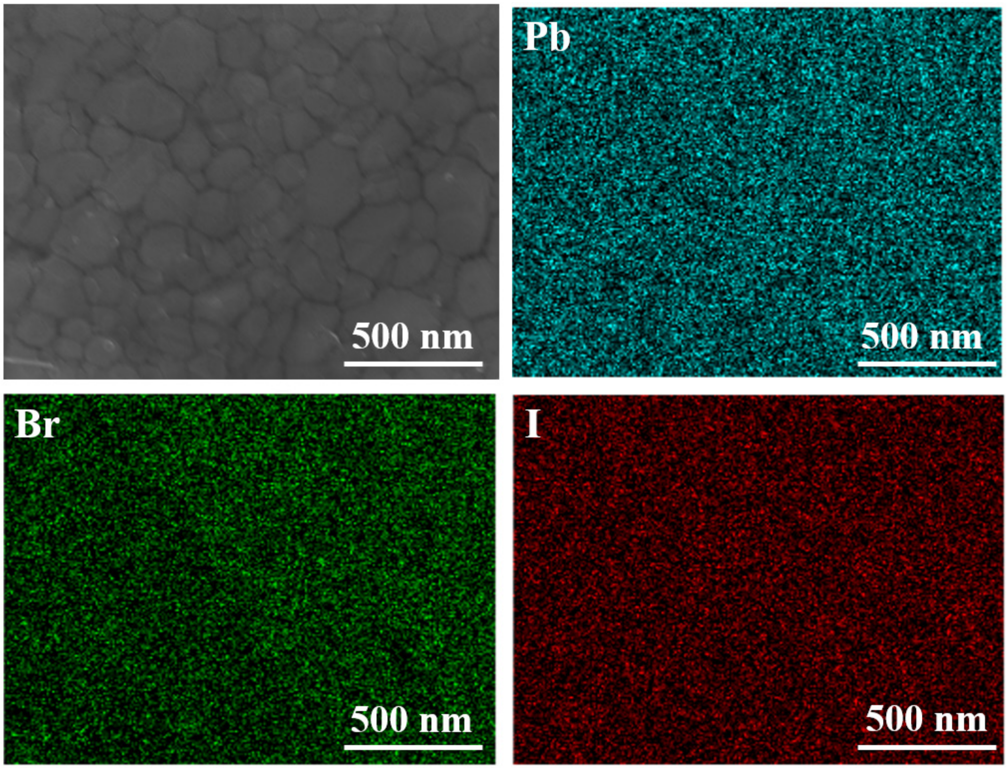


| Element | Weight % | Atom % |
|---|---|---|
| Br | 6.4 | 11.39 |
| I | 56.2 | 62.96 |
| Pb | 37.4 | 25.66 |

**Supplementary Figure 7 | Elemental mapping of the spin-coated $FA_{0.85}MA_{0.15}PbI_{2.55}Br_{0.45}$ perovskite film measured by energy-dispersive X-ray spectroscopy.**

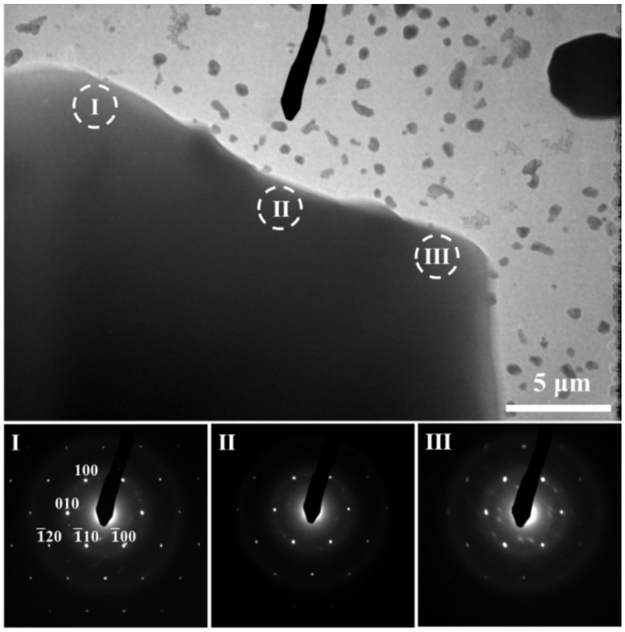


**Supplementary Figure 8 | Three selected area electron diffraction patterns (three lower panels) of $FA_{0.85}MA_{0.15}PbI_{2.55}Br_{0.45}$ perovskite grain with the selected-area size of 2 μm (upper panel).**

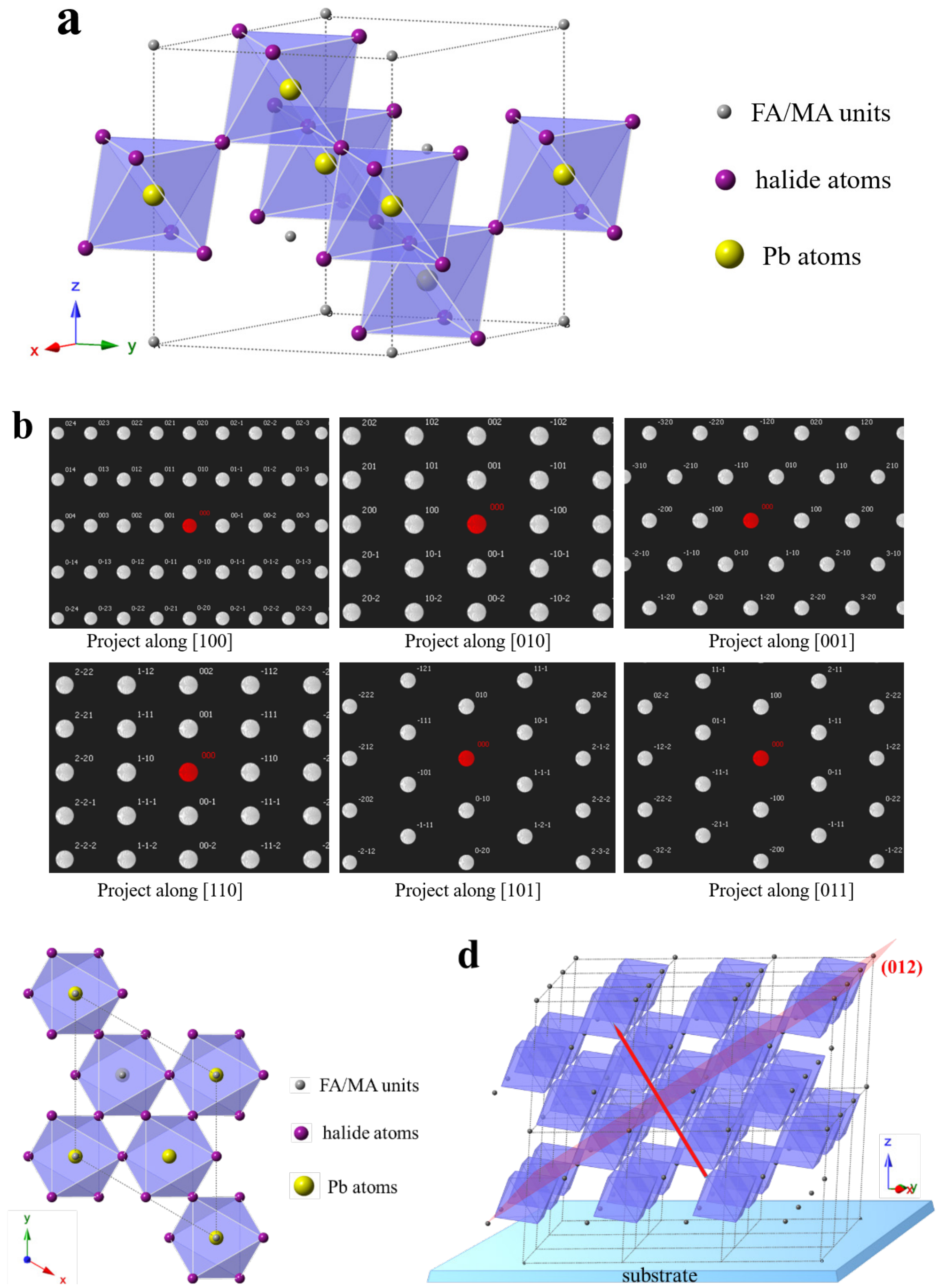


**Supplementary Figure 9 | Crystallographic characterizations of $FA_{0.85}MA_{0.15}PbI_{2.55}Br_{0.45}$ perovskite films casted by meniscus-assisted solution printing.** (**a**) Schematic illustration of a primitive unit cell of the trigonal crystal structure of $FA_{0.85}MA_{0.15}PbI_{2.55}Br_{0.45}$ perovskites. (**b**) Simulated crystal diffraction of a trigonal perovskite crystal with the space group *P3m1*, projected along various axes. (**c**) [001] view of a primitive unit cell of the $FA_{0.85}MA_{0.15}PbI_{2.55}Br_{0.45}$ perovskite. (**d**) Schematic illustration of the ordered, closely packed

corner-sharing lead halide octahedrals along the <012> orientation of $FA_{0.85}MA_{0.15}PbI_{2.55}Br_{0.45}$ perovskites.

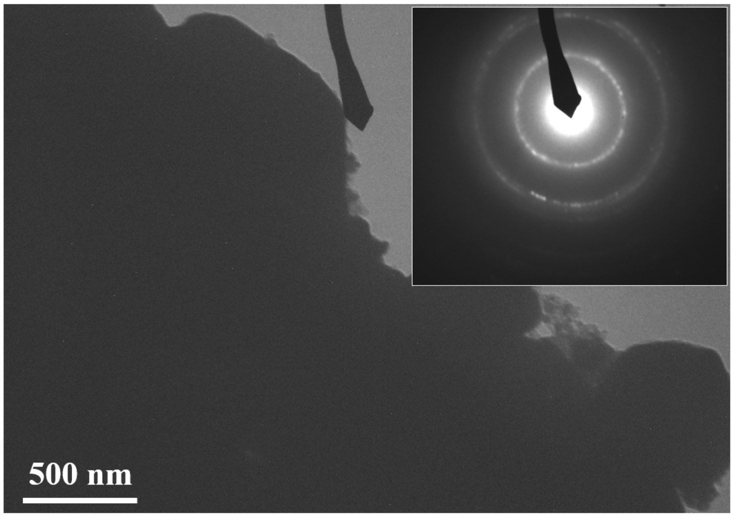


**Supplementary Figure 10 | The scanning electron microscopy image and the selected area electron diffraction pattern of the spin-coated $FA_{0.85}MA_{0.15}PbI_{2.55}Br_{0.45}$ perovskite film.**

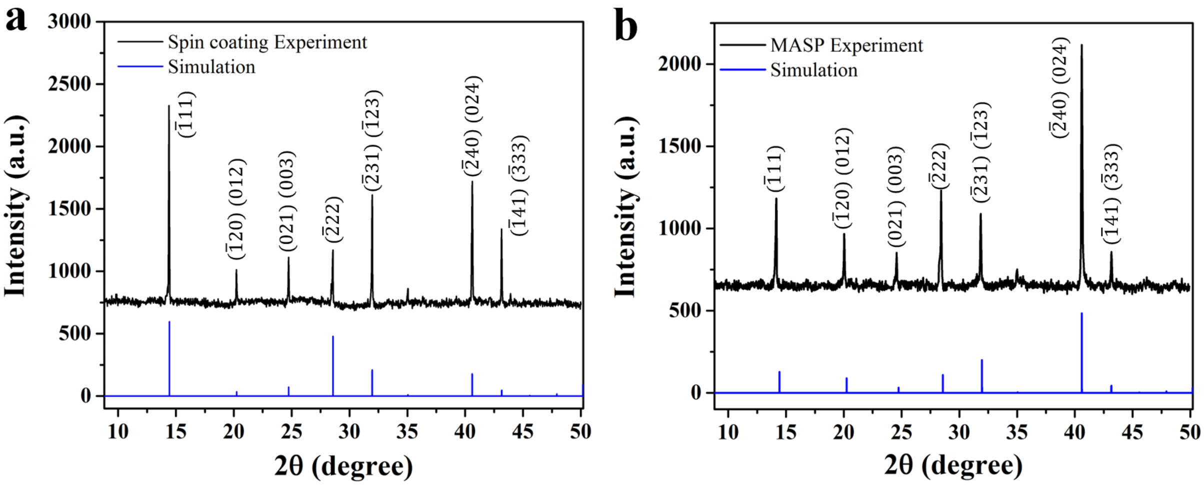


**Supplementary Figure 11 | X-ray diffraction (XRD) characterizations of $FA_{0.85}MA_{0.15}PbI_{2.55}Br_{0.45}$ perovskite films.** Experimental and simulated XRD profiles of (**a**) the $FA_{0.85}MA_{0.15}PbI_{2.55}Br_{0.45}$ perovskite film prepared by spin coating and (**b**) the $FA_{0.85}MA_{0.15}PbI_{2.55}Br_{0.45}$ perovskite film produced by meniscus-assisted solution printing.

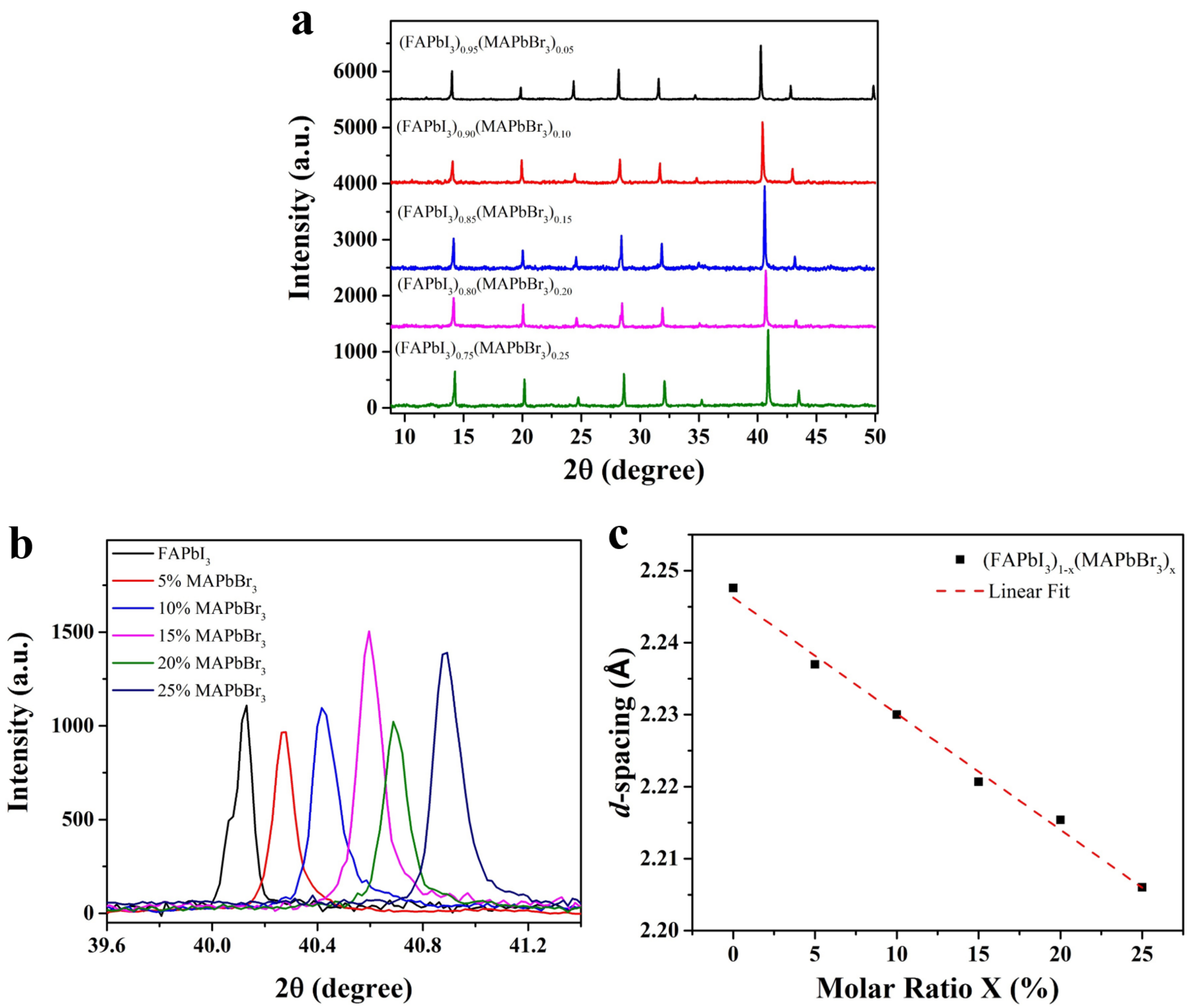


**Supplementary Figure 12 | X-ray diffraction (XRD) characterizations of $FA_{1-x}MA_xPbI_{3(1-x)}Br_{3x}$ perovskite films with varied chemical compositions.** (**a**) XRD profiles of $FA_{1-x}MA_xPbI_{3(1-x)}Br_{3x}$ perovskite films with varied chemical compositions ($0.05 \leq x \leq 0.25$) prepared by MASP. (**b**) The shift of the ($\bar{2}40$)/(024) diffraction peak with an increased molar ratio of $MAPbBr_3$ in $FA_{1-x}MA_xPbI_{3(1-x)}Br_{3x}$ ($0.05 \leq x \leq 0.25$). (**c**) Plot of the *d*-spacing value of $FA_{1-x}MA_xPbI_{3(1-x)}Br_{3x}$ ($0.05 \leq x \leq 0.25$) perovskite films as a function of the molar ratio of $MAPbBr_3$ in $FA_{1-x}MA_xPbI_{3(1-x)}Br_{3x}$.

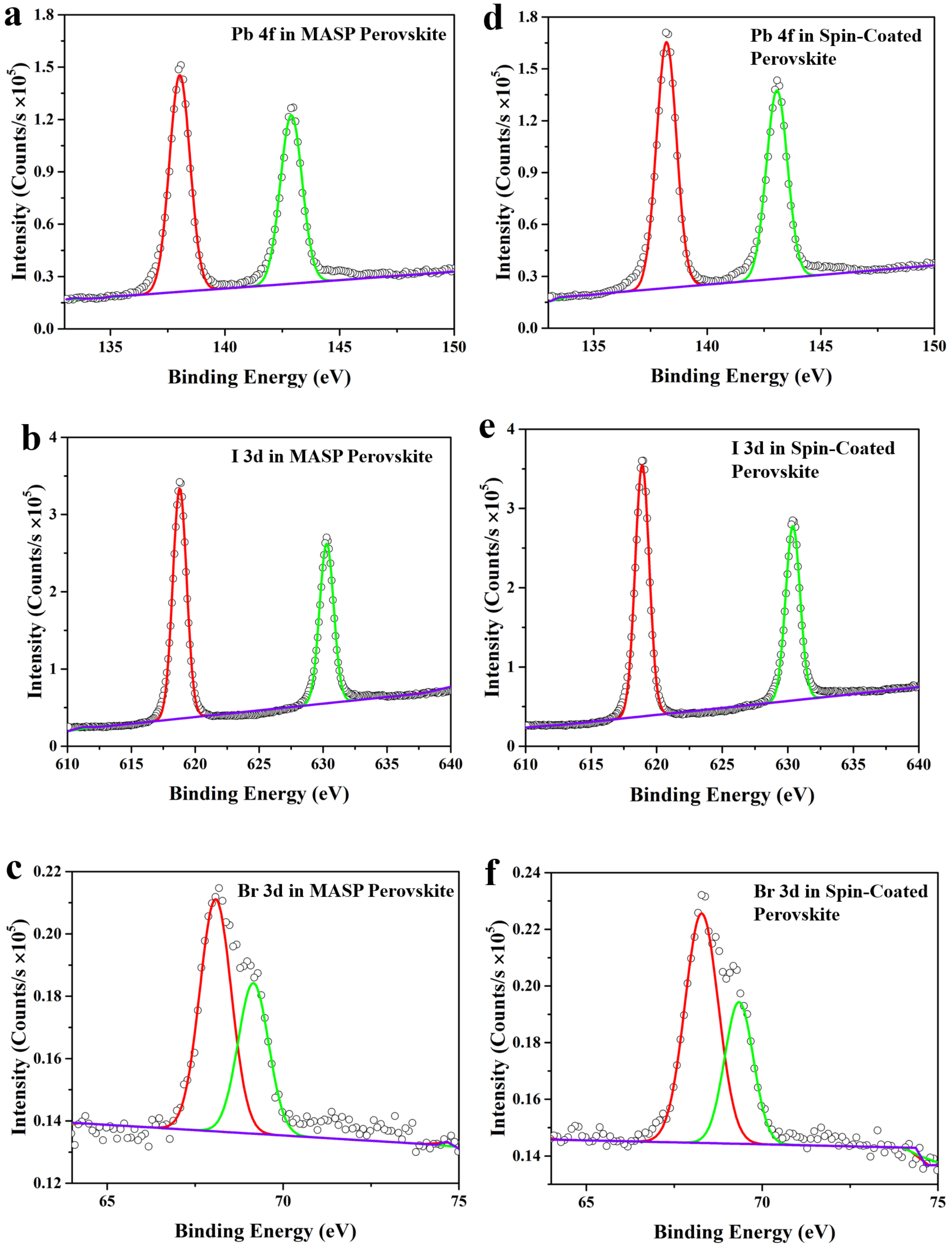


**Supplementary Figure 13 | The chemical composition of $FA_{0.85}MA_{0.15}PbI_{2.55}Br_{0.45}$ perovskite films measured by X-ray photoelectron spectroscopy.** Pb 4f core level spectra of (**a**) meniscus-assisted solution printing (MASP) film, and (**d**) spin-coated film. I 3d core level

spectra of (**b**) MASP film, and (**e**) spin-coated film. Br 3d core level spectra of (**c**) MASP film, and (**f**) spin-coated film.

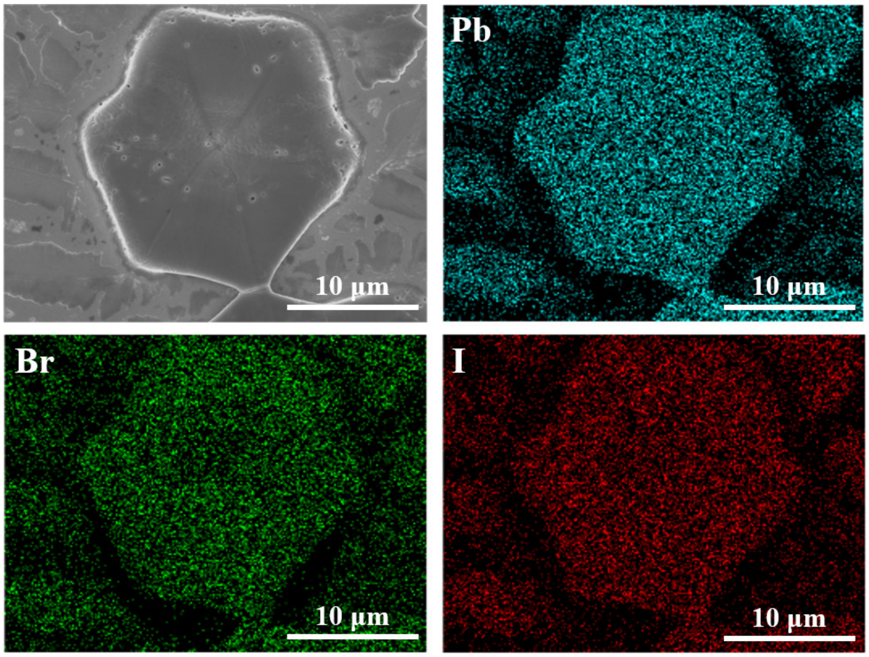


| Element | Weight % | Atom % |
|---|---|---|
| Br | 6.60 | 11.61 |
| I | 58.34 | 64.61 |
| Pb | 35.06 | 23.78 |

**Supplementary Figure 14 | Elemental mapping of the meniscus-assisted solution printed $FA_{0.85}MA_{0.15}PbI_{2.55}Br_{0.45}$ perovskite crystal island.**

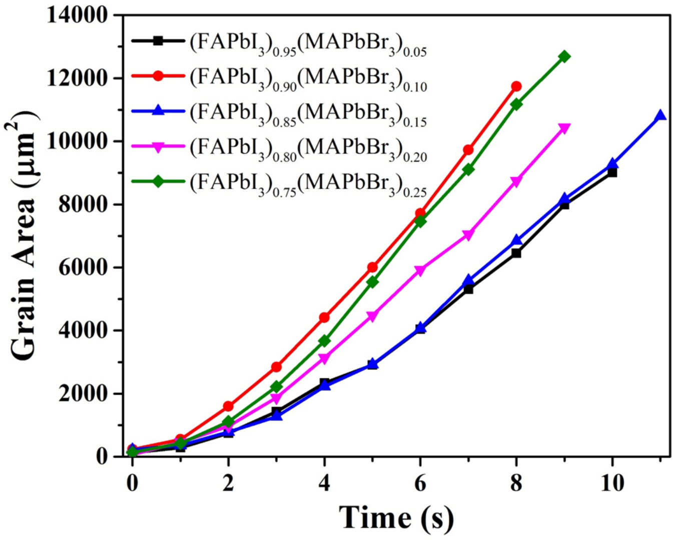


**Supplementary Figure 15 | Plot of the island area of $FA_{1-x}MA_xPbI_{3(1-x)}Br_{3x}$ perovskites with varied chemical compositions ($0.05 \leq x \leq 0.25$) as a function of time.**

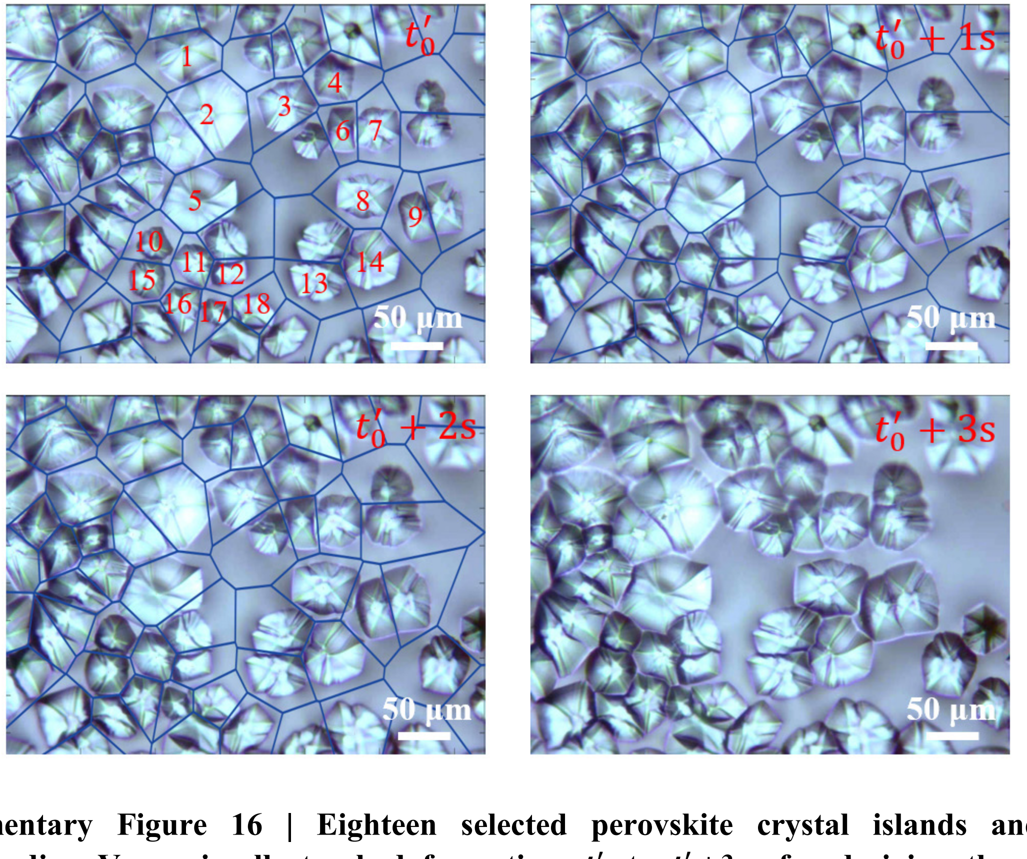


**Supplementary Figure 16 | Eighteen selected perovskite crystal islands and their corresponding Voronoi cells tracked from time $t_0'$ to $t_0'$+*3* s for deriving the average growth rate as a function of the average Voronoi cell area for each island.**

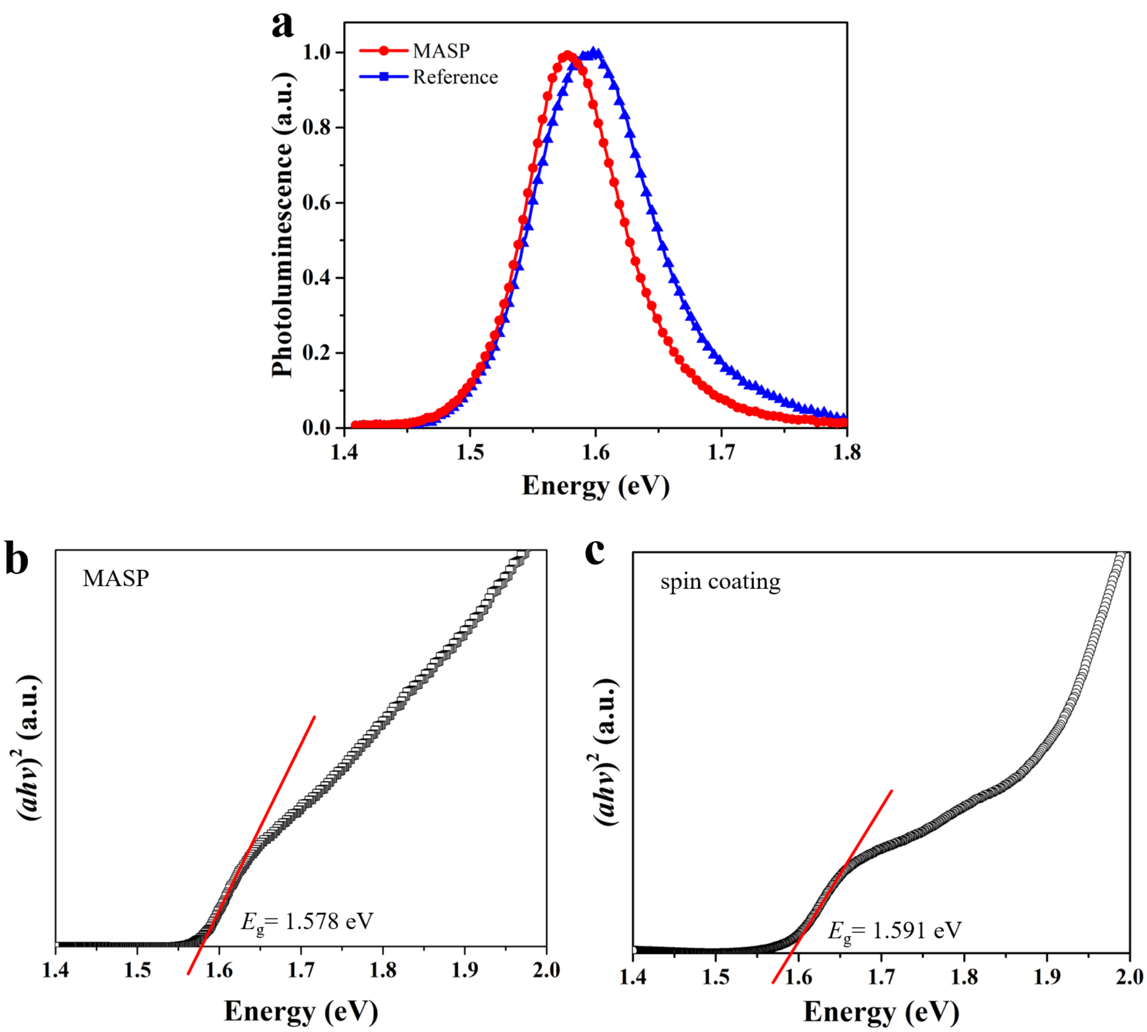


**Supplementary Figure 17 | Photoluminescence and UV-vis characterizations of $FA_{0.85}MA_{0.15}PbI_{2.55}Br_{0.45}$ perovskite films crafted by meniscus-assisted solution printing.** (**a**) Photoluminescence spectra of the $FA_{0.85}MA_{0.15}PbI_{2.55}Br_{0.45}$ perovskite film crafted by MSAP and the reference sample prepared by spin coating. (**b**) The bandgap $E_g$ of $FA_{0.85}MA_{0.15}PbI_{2.55}Br_{0.45}$ perovskite film crafted by MASP estimated from the absorption edge. (**c**) The estimated bandgap $E_g$ of the $FA_{0.85}MA_{0.15}PbI_{2.55}Br_{0.45}$ perovskite film prepared by spin coating.

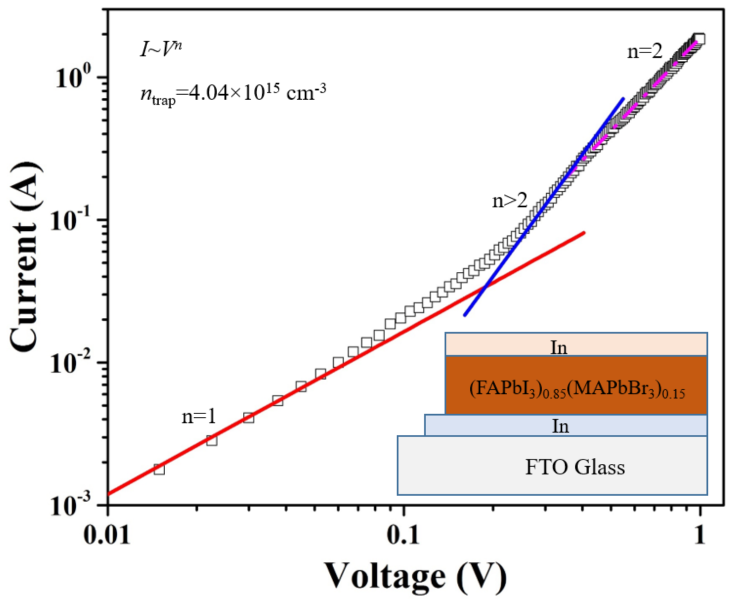


**Supplementary Figure 18 | Current–voltage curve of the perovskite film prepared by spin coating for space charge limited current (SCLC) analysis. The inset illustrates the SCLC architecture.**

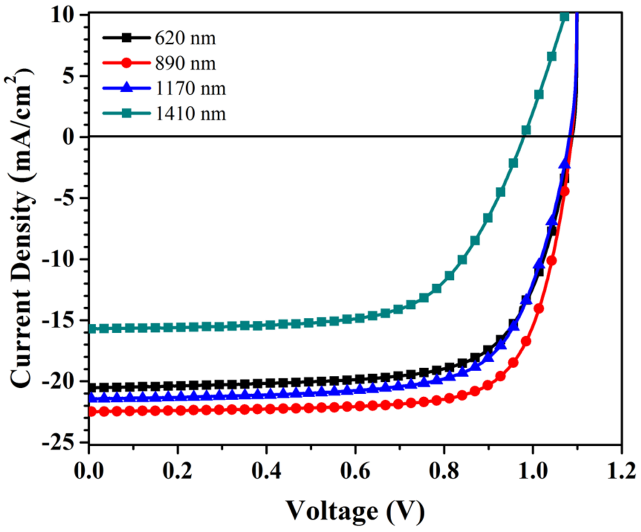


**Supplementary Figure 19 | Current density-voltage curves of $FA_{0.85}MA_{0.15}PbI_{2.55}Br_{0.45}$ perovskite solar cells with different perovskite film thicknesses.**

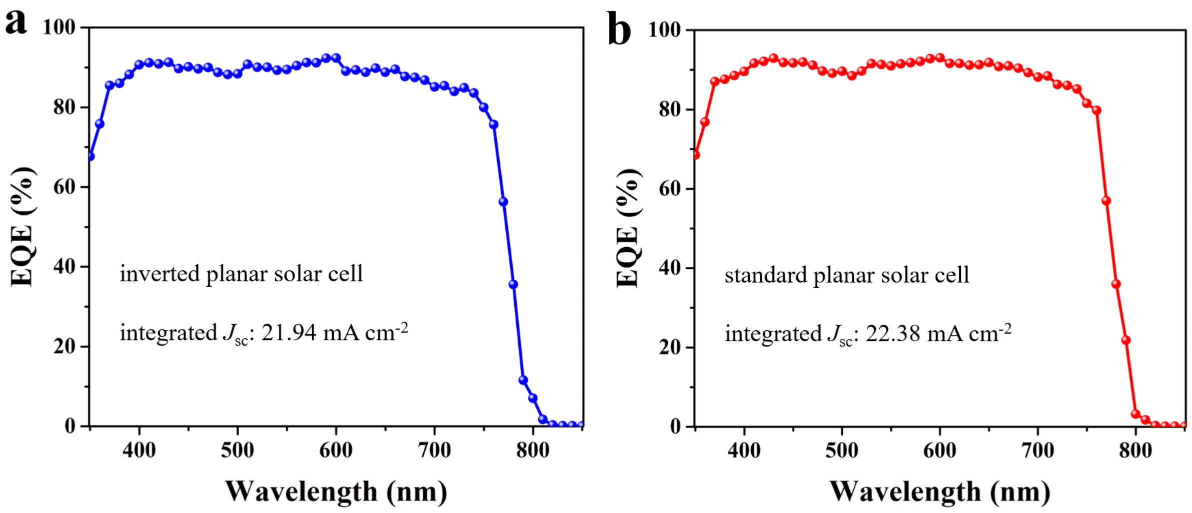


**Supplementary Figure 20 | External quantum efficiency (EQE) characterizations of $FA_{0.85}MA_{0.15}PbI_{2.55}Br_{0.45}$ perovskite solar cells.** The EQE profiles of (**a**) the inverted planar $FA_{0.85}MA_{0.15}PbI_{2.55}Br_{0.45}$ perovskite solar cell fabricated by meniscus-assisted solution printing (MASP) and (**b**) the standard planar $FA_{0.85}MA_{0.15}PbI_{2.55}Br_{0.45}$ perovskite solar cell fabricated by MASP.

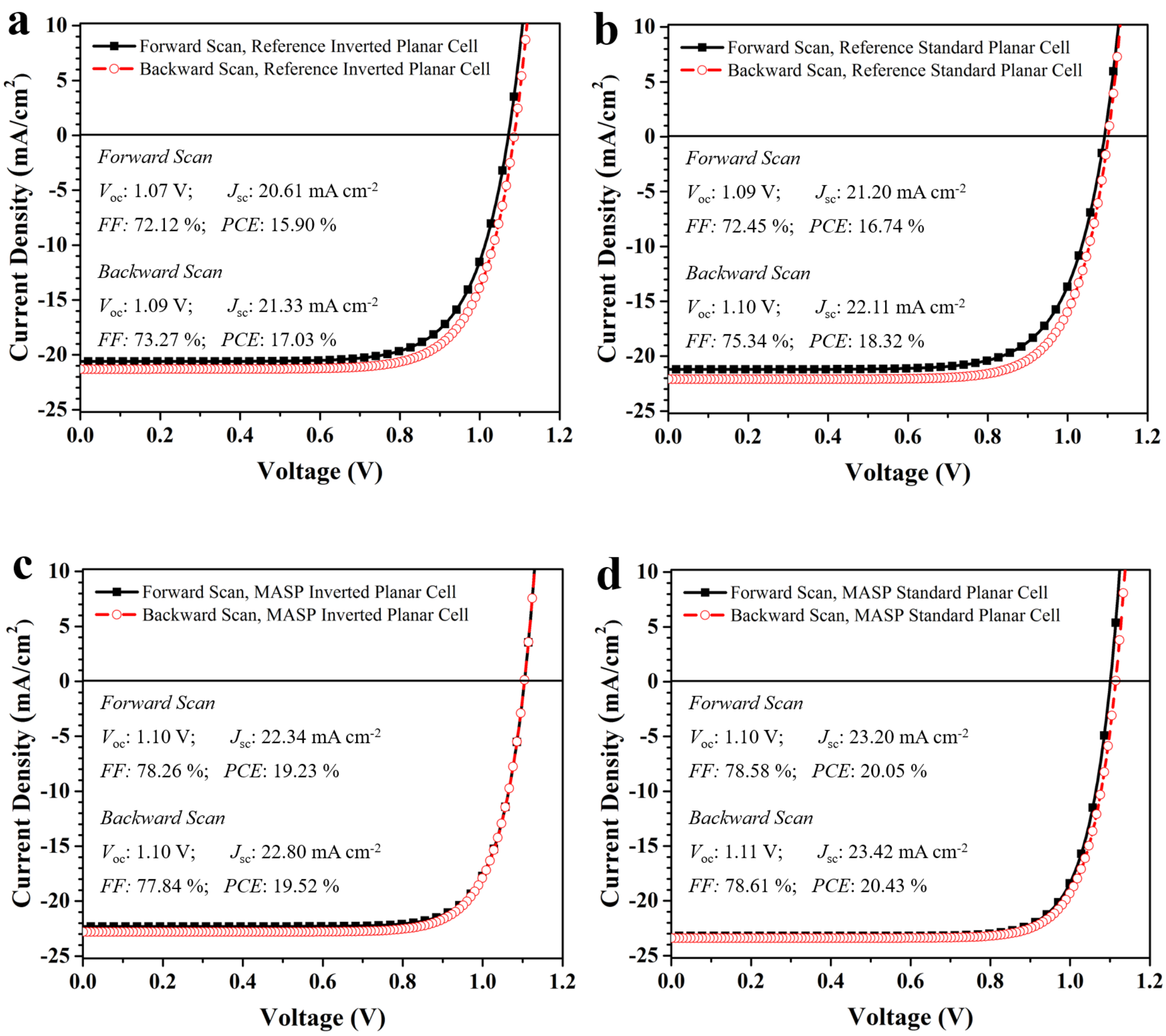


**Supplementary Figure 21 | Current density-voltage (*J*-*V*) measurements of $FA_{0.85}MA_{0.15}PbI_{2.55}Br_{0.45}$ perovskite solar cells.** The *J*-*V* plots scanned in the forward and backward directions: (**a**) the inverted and (**b**) the standard planar $FA_{0.85}MA_{0.15}PbI_{2.55}Br_{0.45}$ perovskite solar cells fabricated by spin coating as references; (**c**) the inverted and (**d**) the standard planar $FA_{0.85}MA_{0.15}PbI_{2.55}Br_{0.45}$ perovskite solar cells fabricated by meniscus-assisted solution printing.

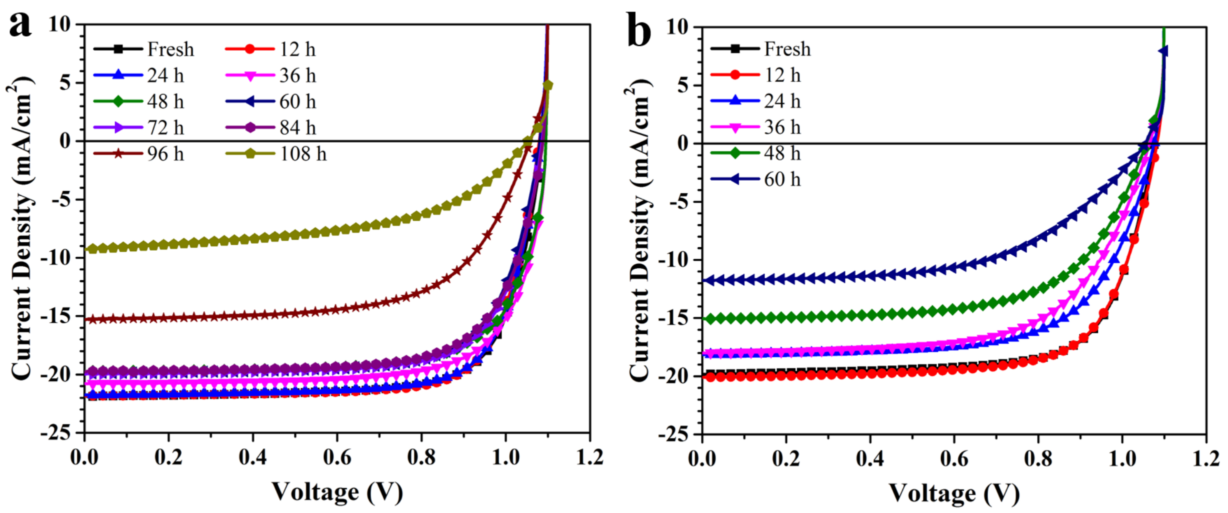


**Supplementary Figure 22 | Current density-voltage (*J-V*) curves during the stability measurement.** The *J-V* curves of (**a**) meniscus-assisted solution printed and (**b**) spin-coated $FA_{0.85}MA_{0.15}PbI_{2.55}Br_{0.45}$ perovskite solar cells. The device stability was measured under continuous AM1.5G solar illumination in ambient environment without encapsulation.

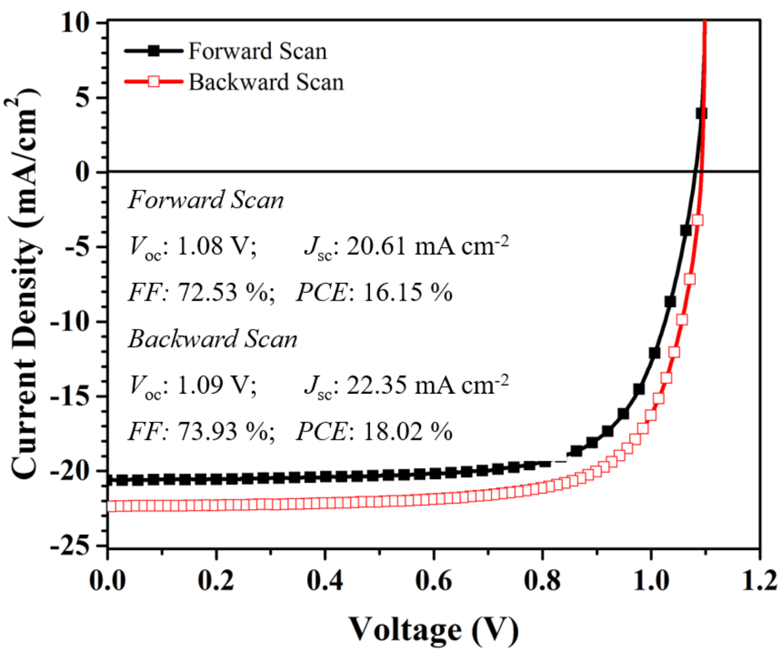


**Supplementary Figure 23 | Current density-voltage curves of the meniscus-assisted solution printed large-area standard planar $FA_{0.85}MA_{0.15}PbI_{2.55}Br_{0.45}$ perovskite solar cells scanned in the forward and backward directions, respectively.**

**Supplementary Table 1 | Photovolatic parameters of $FA_{0.85}MA_{0.15}PbI_{2.55}Br_{0.45}$ perovskite solar cells with different perovskite film thicknesses.**

| Thickness (nm) | $V_{oc}$ (V) | $J_{sc}$ (mA $cm^{-2}$) | *FF* (%) | *PCE* (%) |
|---|---|---|---|---|
| 620 | 1.091 | 20.53 | 70.44 | 15.73 |
| 890 | 1.092 | 22.46 | 74.93 | 18.27 |
| 1070 | 1.083 | 21.45 | 70.65 | 16.37 |
| 1410 | 0.979 | 15.69 | 64.97 | 9.96 |

**Supplementary Table 2 | Summary of photovoltaic parameters from a batch of 10 inverted planar $FA_{0.85}MA_{0.15}PbI_{2.55}Br_{0.45}$ perovskite solar cells fabricated by meniscus-assisted solution printing.**

| Inverted Planar Devices | $V_{oc}$ (V) | $J_{sc}$ (mA $cm^{-2}$) | *FF* (%) | *PCE* (%) |
|---|---|---|---|---|
| 1 | 1.089 | 22.20 | 77.17 | 18.65 |
| 2 | 1.095 | 22.23 | 76.87 | 18.70 |
| 3 | 1.084 | 21.59 | 76.76 | 17.97 |
| 4 | 1.099 | 21.26 | 77.40 | 18.09 |
| 5 | 1.100 | 22.34 | 78.26 | 19.23 |
| 6 | 1.093 | 21.05 | 78.04 | 17.95 |
| 7 | 1.094 | 22.13 | 78.03 | 18.88 |
| 8 | 1.087 | 21.54 | 77.86 | 18.23 |
| 9 | 1.087 | 21.67 | 76.94 | 18.12 |
| 10 | 1.097 | 22.28 | 77.86 | 19.03 |
| Average | 1.092±0.005 | 21.83±0.47 | 77.52±0.56 | 18.49±0.47 |

**Supplementary Table 3 | Summary of photovoltaic parameters from a batch of 10 standard planar $FA_{0.85}MA_{0.15}PbI_{2.55}Br_{0.45}$ perovskite solar cells fabricated by meniscus-assisted solution printing.**

| Standard Planar Devices | $V_{oc}$ (V) | $J_{sc}$ (mA cm$^{-2}$) | *FF* (%) | *PCE* (%) |
|---|---|---|---|---|
| 1 | 1.087 | 22.24 | 77.41 | 18.70 |
| 2 | 1.096 | 22.52 | 78.49 | 19.37 |
| 3 | 1.085 | 22.56 | 78.06 | 19.10 |
| 4 | 1.094 | 22.14 | 78.45 | 19.00 |
| 5 | 1.097 | 22.87 | 77.45 | 19.42 |
| 6 | 1.096 | 22.32 | 78.57 | 19.22 |
| 7 | 1.090 | 22.33 | 77.67 | 18.90 |
| 8 | 1.100 | 23.20 | 78.58 | 20.05 |
| 9 | 1.092 | 22.76 | 77.62 | 19.29 |
| 10 | 1.094 | 22.54 | 77.56 | 19.13 |
| Average | 1.093±0.005 | 22.55±0.32 | 77.98±0.50 | 19.22±0.36 |

**Supplementary Table 4 | Summary of photovoltaic parameters from a batch of 10 inverted planar $FA_{0.85}MA_{0.15}PbI_{2.55}Br_{0.45}$ perovskite solar cells fabricated by spin coating.**

| Inverted Planar Devices | $V_{oc}$ (V) | $J_{sc}$ (mA cm$^{-2}$) | *FF* (%) | *PCE* (%) |
|---|---|---|---|---|
| 1 | 1.044 | 19.42 | 67.62 | 13.71 |
| 2 | 1.041 | 17.08 | 65.59 | 11.66 |
| 3 | 1.066 | 20.47 | 64.96 | 14.17 |
| 4 | 1.041 | 18.97 | 64.50 | 12.74 |
| 5 | 1.050 | 19.19 | 69.33 | 13.96 |
| 6 | 1.067 | 17.76 | 65.26 | 12.37 |
| 7 | 1.061 | 17.65 | 65.51 | 12.27 |
| 8 | 1.070 | 20.61 | 72.12 | 15.90 |
| 9 | 1.052 | 19.91 | 70.48 | 14.77 |
| 10 | 1.039 | 18.78 | 70.92 | 13.84 |
| Average | 1.053±0.012 | 18.98±1.20 | 67.63±2.85 | 13.54±1.29 |

**Supplementary Table 5 | Summary of photovoltaic parameters from a batch of 10 standard planar $FA_{0.85}MA_{0.15}PbI_{2.55}Br_{0.45}$ perovskite solar cells fabricated by spin coating.**

| Standard Planar Devices | $V_{oc}$ (V) | $J_{sc}$ (mA cm$^{-2}$) | *FF* (%) | *PCE* (%) |
|---|---|---|---|---|
| 1 | 1.073 | 20.43 | 72.14 | 15.80 |
| 2 | 1.071 | 18.24 | 69.37 | 13.56 |
| 3 | 1.079 | 20.09 | 67.93 | 14.73 |
| 4 | 1.087 | 20.57 | 67.04 | 15.00 |
| 5 | 1.087 | 20.40 | 71.70 | 15.90 |
| 6 | 1.090 | 21.20 | 72.45 | 16.74 |
| 7 | 1.085 | 19.24 | 69.15 | 14.44 |
| 8 | 1.074 | 19.69 | 69.73 | 14.74 |
| 9 | 1.085 | 20.08 | 72.38 | 15.77 |
| 10 | 1.074 | 18.56 | 70.50 | 14.05 |
| Average | 1.080±0.007 | 19.85±0.93 | 70.24±1.92 | 15.07±0.97 |

**Supplementary Note 1: outward convective flow in meniscus**

The equations of the solvent evaporation and the velocity of the outward convective flow within the meniscus ink can be derived by including the effect of the meniscus geometry.

**(a) Derive a mathematical equation to represent the outline of the meniscus**

The meniscus geometry can be described by a parabolic equation $h=a(R-r)^b$, where $R$ is the distance of the edge of meniscus away from the bulk ink, $r$ is the horizontal distance of the meniscus surface away from the bulk ink, and $h$ is the height of the meniscus at $r$ as shown in Figure 1b. We fit the experimentally measured outline of the meniscus curve with the parabolic equation, giving $a$=0.34, $b$=2.0, and the corresponding fitted line agreed well the experimental meniscus curve (i.e., the red dashed line in Fig. 1b). Thus, we have the mathematical equation to represent the geometric of the meniscus curve as:

$$h = a(R-r)^{\mathrm{b}} \qquad (1)$$

**(b) Calculate the velocity of the outward convective flow based on the meniscus geometry equation**

By applying the lubrication approximation to Navier-Stokes equation, the evolution equation of the local thickness of meniscus edge can be given by: [1]

$$\rho\frac{dh}{dt} = -\rho\frac{1}{r}\frac{d(rhv)}{dr} - J_{\mathrm{e}} \qquad (2)$$

where $r$ is the horizontal distance of the meniscus surface away from the bulk ink, $h$ is the height of meniscus at $r$, $v$ is the velocity of outward convective flow induced by solvent evaporation, $\rho$ is the density of the solvent, and $J_e$ is the flux of solvent evaporating per unit area unit time.[2] Therefore, we have:

$$v = \frac{1}{\rho rh}\int_0^r \left(\rho r\frac{dh}{dt} + J_{\mathrm{e}}r\right)dr \qquad (3)$$

The basic assumptions for calculating the velocity of outward convective flow from Navier-Stokes equation with the lubrication approximation include:[1] (i) vapor recoil is ignored; (ii) Marangoni flow is not induced, because the substrate is heated and maintained at a constant temperature; (iii) $v$ is the height-averaged velocity; and (iv) the shape of the meniscus remains relatively unchanged with time, as the volume of loaded ink is sufficient for completing the printing process. Thus, the $\partial h/\partial t$ term can be neglected. Supplementary Equation 3 can be simplified as follows:

$$v = \frac{1}{\rho rh}\int_0^r J_{\mathrm{e}}r\,dr \qquad (4)$$

In fact, $J_e$ is not a constant and is correlated with the meniscus geometry. The non-equilibrium one-sided (NEOS) model describes the evaporation process of the volatile thin

films. It can also be employed for calculating the evaporation flux of a thin droplet.[3-4] Different from the uniform thickness of thin film, the thickness of thin droplet changes from the center of droplet to the edge of droplet. The basic assumptions for calculating the evaporation flux $J_e$ according to the NEOS model include: (i) the mass flux $J_e$ satisfies the Hertz–Knudsen relation;[5-6] (ii) the temperature and the pressure obeys the Clausis–Clapeyron law;[7] and (iii) it is a reaction-limited evaporation with the interface at non-equilibrium.[4] In this context, the evaporative flux $J_e$ can be given by:[8]

$$J_{\mathrm{e}}(h) = \frac{1}{h+K+W} \qquad (5)$$

where $K$ is the non-equilibrium parameter,[4] $W$ is the thermal effect given by $W = \frac{kd_{\mathrm{s}}}{k_{\mathrm{s}}d_0}$ ($k$ is the liquid thermal conductivity, $k_{\mathrm{s}}$ is the thermal conductivity of substrate, $d_0$ is the liquid thickness, and $d_{\mathrm{s}}$ is the thickness of substrate).

By Supplementary Equation 4 and 5, the outward flow velocity $v$ as a function of the distance $r$ away from the bulk ink can be obtained:

$$v = \frac{1}{\rho r h}\int_0^r \left(\frac{r}{h+K+W}\right) dr \qquad (6)$$

Assuming that $R$ = 400 μm, an example plot of $v$ as a function of the horizontal distance $r$ is shown in Supplementary Figure 1, suggesting that the outward convective flow is sharply accelerated at the edge of meniscus (i.e., 350 μm ≤ $r$ ≤ 400 μm) due to the faster solvent evaporation. We note that we are using a radial model above to describe the outward convective flow towards the meniscus front edge. A 2D form of the realistic meniscus geometry will slow down outwards flow, yet will result in a qualitatively equivalent description (i.e., flow is accelerated towards meniscus front edge).

**Supplementary Note 2: optimal operation window for meniscus-assisted solution printing**

Recently, Janneck et al. have built a physical model to predict the optimal window for the coating speed by estimating the equilibrium front evaporation speed $v_{\mathrm{efe}}$ in the evaporation regime of meniscus coating,[9] in which $v_{\mathrm{efe}}$ is correlated with the substrate temperature $T$, the solvent boiling point $T_{\mathrm{b}}$, and the solvent density $\rho$:

$$v_{\mathrm{efe}} = AV_{\mathrm{m}}T^{0.7}exp\left(-\frac{\Delta S_{\mathrm{vap}}}{R_{\mathrm{i}}}\frac{T_{\mathrm{b}}}{T}\right) \qquad (7)$$

where $V_{\mathrm{m}}$ is the molar volume of solvent, $\Delta S_{\mathrm{vap}}$ is the entropy of vaporization of solvent, $R_{\mathrm{i}}$ is the ideal gas constant, $\Delta S_{\mathrm{vap}}/R_{\mathrm{i}}$ is fixed to 10.5 based on Trouton's rule, and $A$ is 1870 μm $s^{-1}$ mol $mL^{-1}$ $K^{-0.7}$ derived from the fitting of Supplementary Equation 7 with varied solvents. We employed this model to further analyze the front evaporation speed of DMSO as a function of the substrate temperature, the boiling point, and the density in meniscus-assisted solution printing (MASP). The calculated $v_{\mathrm{efe}}$ of DMSO at 60 $^{\circ}$C is 4 μm $s^{-1}$ (Supplementary Figure 5a), the

optimized coating speed $v_c$ we used in MASP was 12 μm $s^{-1}$, thus the normalized coating speed $\bar{v}_c = v_c/v_{efe} = 3$, suggesting that this model may be able to predict the optimal window of the coating speed via estimating the front solvent evaporation rate.

According to the calculated relationship between $v_{efe}$ and $T$ in Supplementary Figure 5a, we can easily tune the optimum speed at different temperatures to make MASP work in its optimal speed window. However, we found that there was also an optimal substrate temperature (i.e., $T = 60 \pm 10$ °C) for depositing uniform $FA_{0.85}MA_{0.15}PbI_{2.55}Br_{0.45}$ perovskite films by MASP. Because DMSO is a high-boiling-point solvent (e.g., $T_b$=189 °C), a low substrate temperature ($T < 50$ °C) usually made DMSO not completely evaporated during the MASP process. Thus, the as-deposited film easily formed cracks and holes due to the evaporation of residual DMSO. These structural defects in perovskite films tend to generate unfavorable charge traps and seriously reduce the efficiencies of charge generation and transport. If the substrate temperature was too high (i.e., $T > 70$ °C), the evaporation of DMSO was largely increased not only at the front edge but also within the bulk meniscus, leading to an increase in solute concentration within the bulk ink, and eventually promoted the direct crystallization of perovskite solutes from the bulk ink.

Therefore, the optimal operation window for the MASP of uniform perovskite films with high power conversion efficiencies (*PCEs*) was fixed at the substrate temperature of 60 °C and the coating speed of 12 μm $s^{-1}$.

In the meniscus coating, the film thickness was typically influenced by the geometry of casting setup, the substrate temperature, the coating speed, the solvent property, and the solute concentration. For the meniscus coating in the Landau-Levich regime, a faster coating speed leads to a thicker film. For the meniscus coating in the evaporative regime, a faster coating speed results in a thinner film.[10] In our work, we fixed our coating process in an optimal operation window as discussed above. In this case, the setup geometry, the substrate temperature, and the coating speed were fixed, and we can adjust the film thickness by varying the perovskite solute concentration. Supplementary Figure 5b shows the film thickness of MASP-deposited $FA_{0.85}MA_{0.15}PbI_{2.55}Br_{0.45}$ perovskite film in relation with the solution concentration.

Through the model analysis and the numerical simulations, Janneck et al. demonstrated that the dependence of the solvent evaporation rate at the meniscus edge $v_{efe}$ on the meniscus geometrical factor $h(\theta)$ was very limited for the droplet length $L$ in the range $4k^{-1} < L < 20k^{-1}$, where $k^{-1}$ is the capillary length. For DMSO, its capillary length at 60 °C is $k^{-1} = \sqrt{\frac{\gamma}{\rho_S g}} = 1.9\,mm$, where $\gamma$ is the surface tension of DMSO (i.e., ~38.84 mN $m^{-1}$ at 60 °C),[11] $\rho_s$ is the DMSO density (i.e., 1.1 g $cm^{-3}$), and $g$ is the gravity. Therefore, the evaporation rate of DMSO was not dependent on the contact angle when the ink droplet length $L$ was controlled in the range of 7.6 mm $< L <$ 38 mm. Janneck et al. studied several different solvents (i.e., dichloromethane,

chloroform, hexane, toluene, anisole, etc.) with different contact angles and boiling points for meniscus coating. They found that all these solvents formed a shallow contact angle ($\theta$ <10 $^{o}$), resulting in similar $h(\theta)$ factors. Accordingly, the contact angle is not expected to largely affect the evaporation rate. In addition, the droplet length of the confined ink could be adjusted by the added ink volume and the separation distance between the two plates. In order to maintain the ink droplet length in the range 7.6 mm < L < 38 mm, we did not largely change the separation distance between the two plates.

**Supplementary Note 3: low-temperature crystallization of perovskites**

The MASP enables the direct formation of high-purity perovskite crystals without the need for high-temperature annealing treatments. Since DMSO has a high boiling point, the lower substrate needed to be heated at 60 $^{o}$C to accelerate the evaporation of DMSO, which in turn generated a driving force for the outward convective flow toward the edge of meniscus, thus forming a supersaturation phase. The trigonal perovskite phase was then directly formed in $FA_{1-x}MA_xPbI_{3(1-x)}Br_{3x}$ ($0.05 \leq x \leq 0.25$) with varied chemical compositions, in which no unpurified phases appeared and all films exhibited preferred orientation along the <$\bar{1}$20>/<012> direction as shown in Supplementary Figure 12a. In addition, the position of ($\bar{2}$40)/(024) diffraction peak gradually shifted to large $2\theta$ with the increased molar ratio of $MAPbBr_3$ in $FA_{1-x}MA_xPbI_{3(1-x)}Br_{3x}$ (Supplementary Figure 12b), indicating that the *d*-spacing of ($\bar{2}$40)/(024) plane was decreased with the increased molar ratio of $MAPbBr_3$. The *d*-spacing value and the molar ratio of $MAPbBr_3$ followed a linear relationship as shown in Supplementary Figure 12c.

**Supplementary Method 1: selected area electron diffraction**

The single crystal diffraction of a trigonal perovskite crystal with the space group *P3m1* was simulated using CrystalMaker SingleCrystal™. The lattice parameters *a*, *b*, and *c* are determined from the *d*-spacing values of the related (*hkl*) directions according to the relationship:

$$d_{hkl} = \frac{1}{\sqrt{\frac{4}{3}\left(\frac{h^2+hk+k^2}{a^2}\right)+\left(\frac{l}{c}\right)^2}} \quad \text{(S8)}$$

The lattice parameters for the MASP-deposited $FA_{0.85}MA_{0.15}PbI_{2.55}Br_{0.45}$ perovskite are: $a = b =$ 8.8533 Å, $c$ = 10.8380 Å, $\alpha = \beta = 90^o$, $\gamma = 120^o$. A primitive unit cell of the trigonal crystal structure of $FA_{0.85}MA_{0.15}PbI_{2.55}Br_{0.45}$ perovskite is illustrated in Supplementary Figure 9a.[12] The simulated diffraction patterns projected along various axes are shown in Supplementary Figure 9b. The selected area electron diffraction pattern of the MASP-enabled $FA_{0.85}MA_{0.15}PbI_{2.55}Br_{0.45}$ perovskite film in the inset of Figure 1d matched with the simulation pattern projected along [001] axis, indicating that the (110) plane of the trigonal perovskite crystal was parallel to the substrate. The unit cell of the $FA_{0.85}MA_{0.15}PbI_{2.55}Br_{0.45}$ trigonal crystal viewed along the [001]

axis is illustrated in Supplementary Figure 9c.[12] Moreover, Supplementary Figure 9d depicts that the corner-sharing lead halide octahedral can form a long-range ordered network along the <012> orientation, which is beneficial for charge transport throughout the crystal film.

## Supplementary Method 2: Rietveld refinement XRD simulation

The X-ray diffraction (XRD) profiles of $FA_{0.85}MA_{0.15}PbI_{2.55}Br_{0.45}$ perovskite film prepared by MASP and the reference sample prepared by spin coating can be readily indexed with the Rietveld refinement simulation of a trigonal perovskite crystal with the space group $P3m1$.[13] The experimental XRD peaks of the spin-coated film agreed well with the Rietveld refinement simulation (Supplementary Figure 11a), in which the $(\bar{1}11)$ peak had the highest intensity as observed in previous work.[14-15] For the $FA_{0.85}MA_{0.15}PbI_{2.55}Br_{0.45}$ perovskite film crafted by MASP, the intensity of $(\bar{2}40)$/(024) peak was significantly increased, suggesting that the MASP film exhibited preferred crystal orientation along the $<\bar{1}20>$/<012> direction. The March distribution function is often used for simulating preferred orientation in the Rietveld refinement:[13]

$$P_{\mathrm{k}} = (r^2 cos^2 a_{\mathrm{k}} + r^{-1} sin^2 a_{\mathrm{k}})^{-\frac{3}{2}} \tag{9}$$

where $P_{\mathrm{k}}$ is the modificaion of the diffraction peak intensity due to the preferred orientation, $\alpha_{\mathrm{k}}$ is the angle between the preferred orientation vector and the normal to the planes, and $r$ is the refinable parameter in the Rietveld refinement. For $r = 1$, there is no prefererd orientation in the crystal, and the simulated intensities of diffraction peaks are shown in Supplementary Figure 11a. When we set the preferred orientaion at the $<\bar{1}20>$/<012> direction with $r = 0.47$, the simulated intensities of diffraction peaks agreed well with the experimental result shown in Supplementary Figure 11b, implying the preferred orientation in the MASP-deposited perovskite film.[16]

## Supplementary Method 3: UV-vis and PL spectra

The optical bandgap $E_{\mathrm{g}}$ of perovskite films was estimated from the Tauc formula:[17]

$$(ahv)^{1/n} = A(hv - E_{\mathrm{g}}) \tag{10}$$

where $a$ is the absorption coefficient, $h$ is Planck's constant, $v$ is frequency, $A$ is constant, $n = 1/2$ for direct allowed transitions. The $E_{\mathrm{g}}$ was derived from the Tauc plot of the perovskite films (i.e., $(ahv)^2$ ~ photon energy $hv$). The increased slope of the UV-Vis absorption edge with the photon energy forms an Urbach tail, which is correlated with the energy transition between the density-of-states tails in the valence band and those in the conduction band. The Urbach behavior of the absorption edge can be described as:[18]

$$\alpha = \alpha_0 \cdot \exp\left[\frac{\sigma(hv-E_0)}{kT}\right] = \alpha_0 \cdot \exp\left[\frac{hv-E_0}{E_U}\right] \tag{11}$$

where $\sigma$ is the steepness parameter of the absorption edge, $E_U = \Delta(hv)/\Delta(\ln a)$ is the Urbach energy, which characterizes the smearing degree of absorption edge caused by compositional and structural disordering.[19]

**Supplementary Method 4: space charge-limited current devices**

The space charge-limited current devices were fabricated and evaluated to estimate the charge trap density in perovskite films. The measured current-voltage curve follows the Mott-Gurney's square law:[15]

$$J_D = \frac{9\varepsilon\varepsilon_0\mu V_b^2}{8L^3} \tag{12}$$

where $J_D$ is the dark current, $\varepsilon$ is the relative dielectric constant, $\varepsilon_0$ is the vacuum permittivity, $\mu$ is the charge carrier mobility, $V_b$ is the applied bias voltage, and $L$ is the film thickness. The charge trap density $N_{trap}$ was derived from the trap-filled limited voltage $V_{TFL}$:[20]

$$V_{TFL} = \frac{eN_{trap}L^2}{9\varepsilon\varepsilon_0} \tag{13}$$

The charge trap density of the spin-coated $FA_{0.85}MA_{0.15}PbI_{2.55}Br_{0.45}$ perovskite film was estimated to be ~$4.04\times10^{15}$ $cm^{-3}$, which was comparable with that in previous work.[15]

## Supplementary References

1. Fischer, B. J., Particle Convection in an Evaporating Colloidal Droplet. *Langmuir.* **18,** 60-67 (2002).
2. Xu, J.; Xia, J.; Hong, S. W.; Lin, Z.; Qiu, F.; Yang, Y., Self-Assembly of Gradient Concentric Rings via Solvent Evaporation from a Capillary Bridge. *Phys. Rev. Lett.* **96,** 066104 (2006).
3. Oron, A.; Davis, S. H.; Bankoff, S. G., Long-scale evolution of thin liquid films. *Rev. Mod. Phys.* **69,** 931-980 (1997).
4. Murisic, N.; Kondic, L., On evaporation of sessile drops with moving contact lines. *J. Fluid. Mech.* **679,** 219-246 (2011).
5. Knudsen, M., Die maximale verdampfungsgeschwindigkeit des quecksilbers. *Ann. Phys-berlin.* **352,** 697-708 (1915).
6. Plesset, M. S.; Prosperetti, A., Flow of vapour in a liquid enclosure. *J. Fluid. Mech.* **78,** 433-444 (1976).
7. Atkin, P.; de Paula, J., Atkins' physical chemistry. *WH Freeman and Company Books* (2006).
8. Burelbach, J. P.; Bankoff, S. G.; Davis, S. H., Nonlinear stability of evaporating/condensing liquid films. *J. Fluid. Mech.* **195,** 463-494 (1988).
9. Janneck, R.; Vercesi, F.; Heremans, P.; Genoe, J.; Rolin, C., Predictive Model for the Meniscus-Guided Coating of High-Quality Organic Single-Crystalline Thin Films. *Adv. Mater.* **28,** 8007-8013 (2016).
10. Le Berre, M.; Chen, Y.; Baigl, D., From Convective Assembly to Landau−Levich Deposition of Multilayered Phospholipid Films of Controlled Thickness. *Langmuir.* **25,** 2554-2557 (2009).
11. Lu, P.; Zhao, G. J.; Zhang, X. L.; Yin, J. G.; Bao, J. F., Measurement and Prediction on the Surface Properties of Dimethyl Sulfoxide/Water Mixtures. *Chem. Res. Chinese. U.* **32,** 100-105 (2016).
12. Stoumpos, C. C.; Malliakas, C. D.; Kanatzidis, M. G., Semiconducting Tin and Lead Iodide Perovskites with Organic Cations: Phase Transitions, High Mobilities, and Near-Infrared Photoluminescent Properties. *Inorg. Chem.* **52,** 9019-9038 (2013).
13. Will, G., *Powder Diffraction: The Rietveld Method and the Two Stage Method to Determine and Refine Crystal Structures from Powder Diffraction Data*. Springer Science & Business Media: 2006.
14. Jeon, N. J.; Noh, J. H.; Yang, W. S.; Kim, Y. C.; Ryu, S.; Seo, J.; Seok, S. I., Compositional engineering of perovskite materials for high-performance solar cells. *Nature* **517,** 476-+ (2015).
15. Han, Q. F.; Bae, S. H.; Sun, P. Y.; Hsieh, Y. T.; Yang, Y.; Rim, Y. S.; Zhao, H. X.; Chen, Q.; Shi, W. Z.; Li, G.; Yang, Y., Single Crystal Formamidinium Lead Iodide (FAPbI(3)): Insight into the Structural, Optical, and Electrical Properties. *Adv. Mater.* **28,** 2253-2258 (2016).
16. Dollase, W. A., Correction of intensities for preferred orientation in powder diffractometry: application of the March model. *J. Appl. Crystallogr.* **19,** 267-272 (1986).
17. Tauc, J.; Menth, A.; Wood, D. L., Optical and Magnetic Investigations of the Localized States in Semiconducting Glasses. *Phys. Rev. Lett.* **25,** 749 (1970).
18. Studenyak, I.; Kranjec, M.; Kurik, M., Urbach Rule in Solid State Physics. *Int. J. Opt. Appl.* **4,** 76-83 (2014).
19. John, S.; Soukoulis, C.; Cohen, M. H.; Economou, E. N., Theory of Electron Band Tails and the Urbach Optical-Absorption Edge. *Phys. Rev. Lett.* **57,** 1777-1780 (1986).
20. Stranks, S. D.; Eperon, G. E.; Grancini, G.; Menelaou, C.; Alcocer, M. J. P.; Leijtens, T.; Herz, L. M.; Petrozza, A.; Snaith, H. J., Electron-Hole Diffusion Lengths Exceeding 1 Micrometer in an Organometal Trihalide Perovskite Absorber. *Science* **342,** 341-344 (2013).
